# Supplementary material for: Quantum evidence of nonlocal consciousness during clinical death
Source: Innovation (Camb). 2026 Mar 25;7(9):101355. doi: 10.1016/j.xinn.2026.101355 (PMC13556340; doi:10.1016/j.xinn.2026.101355)
Supplement: Supplementary file 1 [file mmc1.pdf]

The Innovation, Volume *7*

Supplemental Information

Quantum evidence of nonlocal consciousness during clinical death

Álex Escolà-Gascón, Kenneth Drinkwater, Andrew Denovan, Neil Dagnall, and Julián Benito-León

The Innovation, Volume *7*

Supplemental Information

Quantum evidence of nonlocal consciousness during clinical death

Álex Escolà-Gascón, Kenneth Drinkwater, Andrew Denovan, Neil Dagnall, and Julián Benito-León

Appendix

1. Operational summary of the applied controls

As outlined in the preceding subsections, the design of this study successfully controlled for the following types of bias:

1) Experimenter bias: controlled through the implementation of a double-blind protocol.
2) False memory fabrication bias: addressed by testing the parametric hypothesis concerning the mean number of correct responses in groups A and B. If this type of bias was absent, one would expect the equality hypothesis to hold, such that the difference in means between groups A and B would be zero (H_0: $\mu^A - \mu^B = 0$).
3) Unconscious response bias: addressed by comparing the fit between the distributions of correct responses in the experimental group (A) and control group B, which was exposed to emotionally neutral auditory stimuli. Unlike the previous hypothesis, this analysis focused on the degree of alignment between the response patterns across groups A and B. If no adequacy bias was present, then no significant discrepancies should appear between the two distributions.
4) Causal interdependence bias: controlled by employing random assignment of patients to experimental groups. Random assignment allows for isolation and causal inference, minimizing the risk that confounding variables generate non-random effects (i.e., patterned responses) in the data.

Having accounted for the biases listed above, in order to consider whether NDEs genuinely reflect a transition from quantum effects to macroscopic reality, it becomes necessary to assess the extent to which the results of groups A and B diverged from those of group C. This can be addressed using parametric hypothesis testing on the means and variances across the groups. In the case of variance related to quantum effects, the Fisher-Escolà distribution was applied, via computation of the *Q* statistic. Figure A1 summarizes the group distributions and the experimental conditions assigned to each.

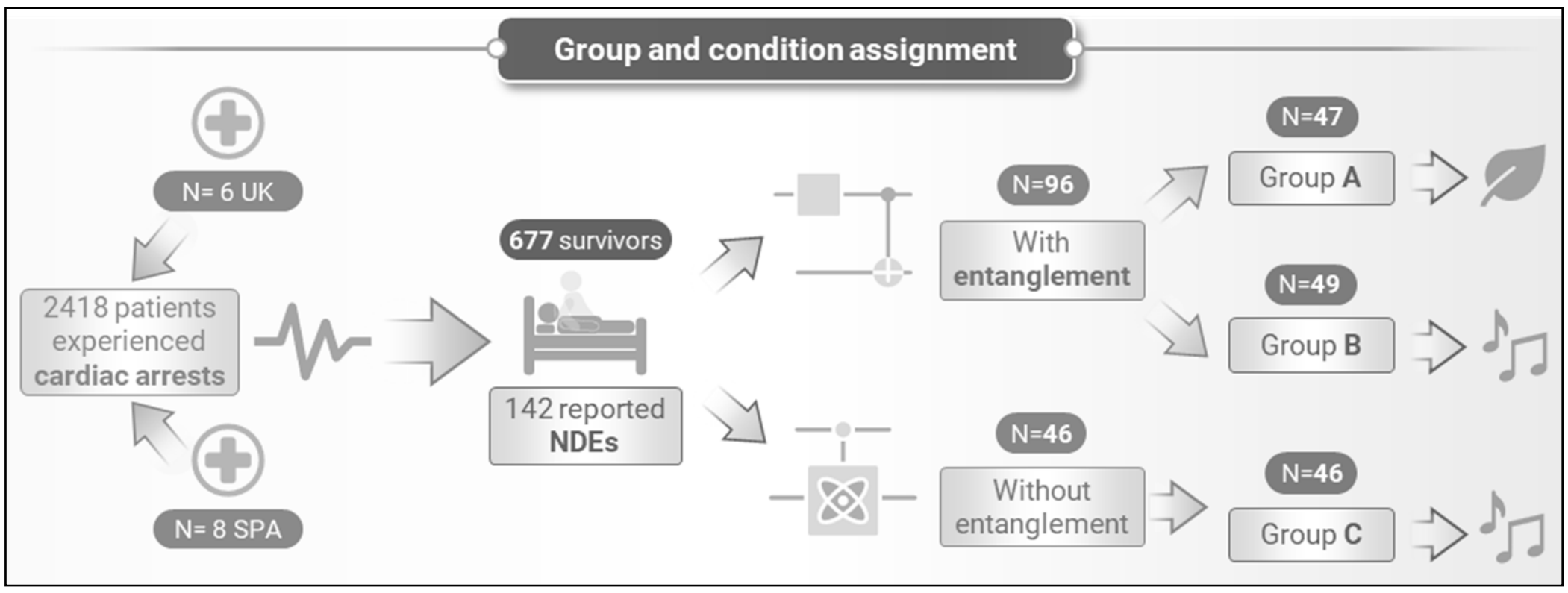

Figure A1. Process diagram for tracking, selecting, and distributing cases. It also details the three groups in this research design, the treatment conditions, and the quantum circuits used. Leaf icon= natural sounds; music icon= neutral sounds; NDE= near-death experience; SPA= Spain; and UK= United Kingdom.

Research title
Quantum evidence of nonlocal consciousness during clinical death

Authors
Álex Escolà-Gascón[1]*
Kenneth Drinkwater[2]
Andrew Denovan[3]
Neil Dagnall[2]
Julián Benito-León[4,5,6,7]*

[1]Department of Quantitative Methods and Statistics, Comillas Pontifical University, established by the Holy See, Vatican City State.
[2]Faculty of Health, Psychology and Social Care, Manchester Metropolitan University, Manchester, United Kingdom.
[3]School of Psychology, Liverpool John Moores University, Liverpool, United Kingdom.
[4]Department of Neurology, 12 de Octubre University Hospital, Madrid, Spain.
[5]Group of Neurodegenerative Diseases, Hospital Universitario 12 de Octubre Research Institute (imas12), Madrid, Spain.
[6]Centro de Investigación Biomédica en Red sobre Enfermedades Neurodegenerativas (CIBERNED), Madrid, Spain.
[7]Department of Medicine, Faculty of Medicine, Complutense University, Madrid, Spain.

***Corresponding authors**
Prof. Álex Escolà-Gascón, Ph.D., Department of Quantitative Methods and Statistics, Comillas Pontifical University, established by the Holy See, Vatican City State (office located at 23 Alberto Aguilera Street, 28015 Madrid, Spain).

Prof. Dr. Julián Benito-León, Department of Neurology, 12 de Octubre University Hospital, Madrid, Spain.

Emails
aescola@icade.comillas.edu
julianbe@ucm.es

2. Composition, illustration, and technical specifications of the quantum and classical circuits

Figures A2 and A3 in this Appendix provide the detailed graphical representations of the circuits employed in the study.

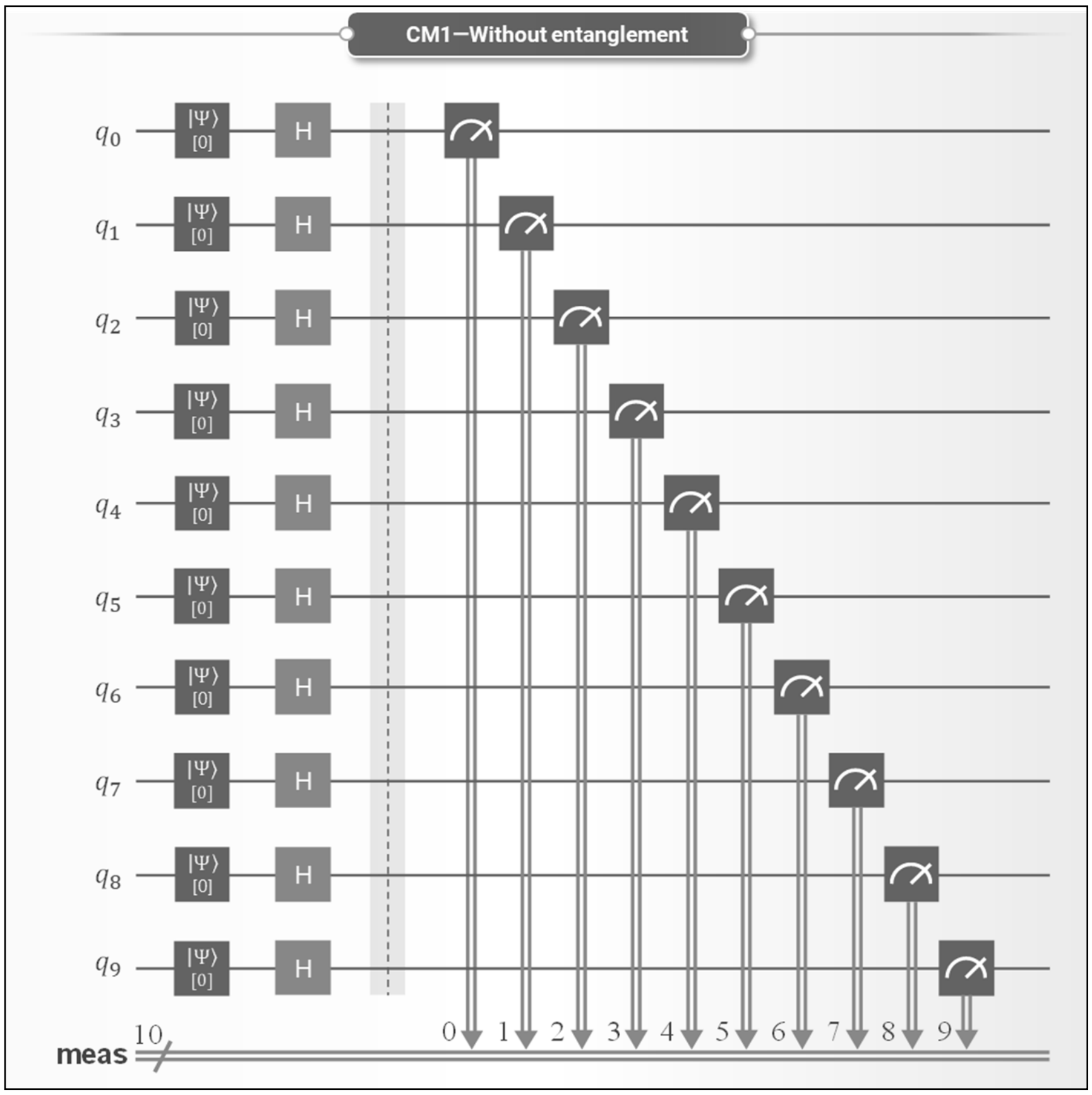

Figure A2. CM1 circuit of the control group, with no entanglement.
The Hadamard gates create superpositions that lead to fully randomized collapses.

Methodological note

The operational conditions under which these circuits were implemented are detailed in the main manuscript. In addition, the reported values of the Pauli operators correspond to sample-based estimations derived by averaging across the full set of circuit executions, in accordance with the specified sample size. The mathematical and analytical procedures employed are likewise described in detail in the main text. Here, meas 10 denotes the ten-dimensional classical measurement register, whereas labels 0 – 9 identify the corresponding classical register indices rather than fixed measurement outcomes. This summary is provided to offer a clearer overview of the scope and complexity of the analyses conducted.

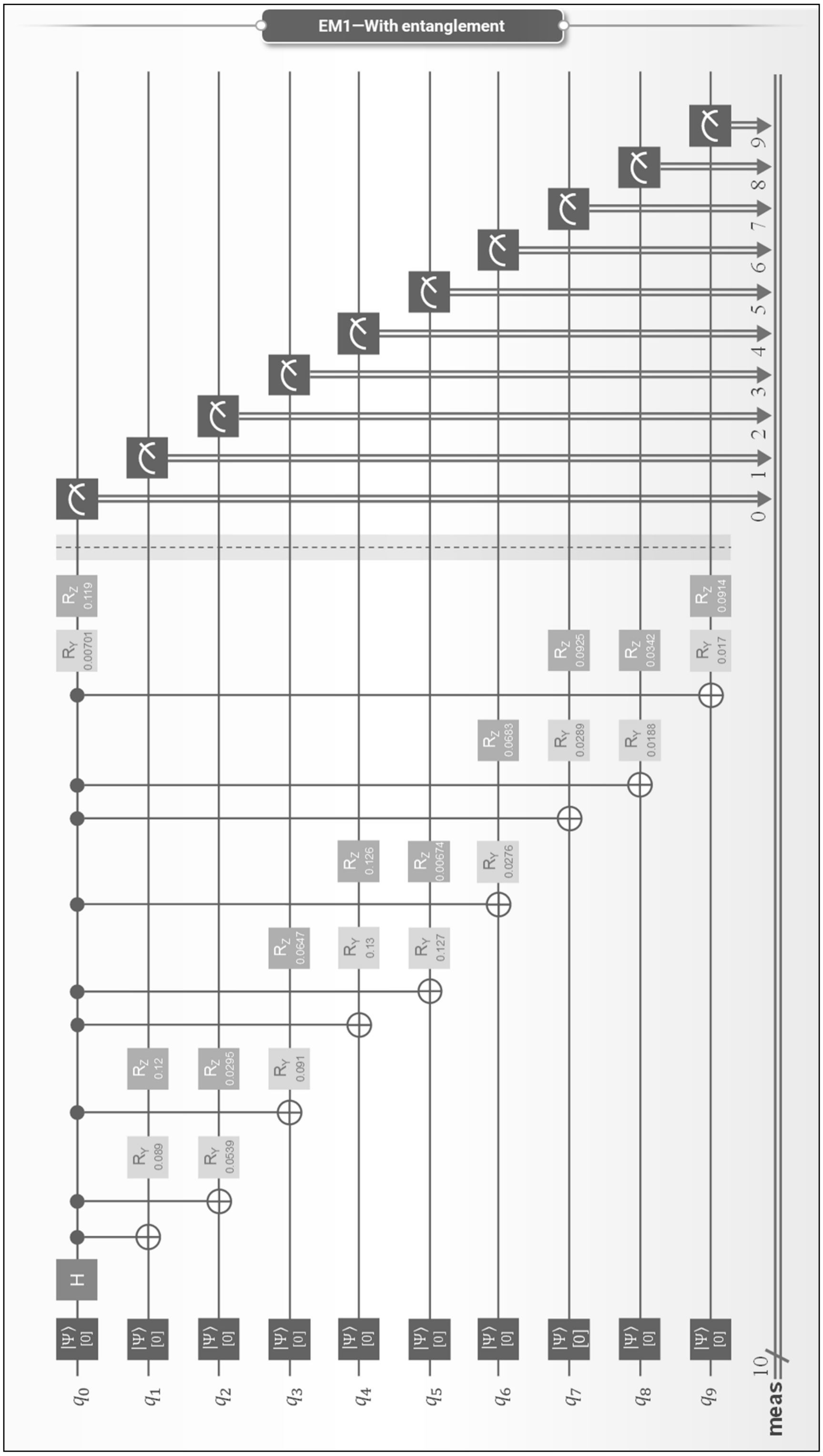

Figure A3. EM1 circuit for experimental group A, where qubits are systematically entangled using CNOT gates to generate nonlocal correlations. Rotational gates were incorporated to prevent collapse invariance.

3. Summary of hypotheses, measurements, and applied techniques

Given the extensive number of analyses and tests conducted in this study, Table A1 summarizes the relationships and correspondences among the research questions, the objects of analysis, and the mathematical-analytical tests employed. This summary is intended to provide a clearer overview of the complexity of the analyses performed.

Table A1.

Correspondences among the intended effects, the objects analyzed in relation to those effects, and the types of statistical and mathematical tests employed.

Field of analysis	Effects under investigation	Support	Types of mathematical and statistical tests applied
Medical analysis	Biomarkers associated with near-death experiences (NDEs).	Markers specified in Table 2 and scales related to NDEs.	Multiple linear regression analysis and econometric modeling.
	Biomarkers of conscious awareness during clinical death.	Average markers specified in Table 2.	Multifactorial analysis of variance, using Fisher's *F* distribution.
Quantum analysis related to consciousness	Quantum properties involved in stimulus configuration during exposure.	Density matrices obtained from the IBM supercomputer backend.	Evaluation of Mermin's GHZ inequality violations, quantum concurrence computation, and von Neumann entropy analysis.
	Empirical verification of the entanglement transition effect.	Comparison of variances and accuracy levels in the perceptual test	Multifactorial analysis of variance, using Fisher's *F* distribution.
	Gradation of the entanglement transition effect.	Explained variance using the Fisher-Escolà *Q* statistic.	Incremental analysis using the Fisher-Escolà *Q* probability distribution.
Experimental and control analysis	False memory formation biases.	Mean comparison (groups A and B) of correct responses in the perceptual test.	Multifactorial analysis of variance, using Fisher's F distribution
	Response and expectation biases.	Analysis of response sequence distributions in the perception tests.	Goodness-of-fit analysis using the *Overlap Coefficient* (OVL) and the Kolmogorov–Smirnov statistical test.
	Psychotic symptomatology and hallucinations biases.	Psychotic hallucination scale, NDE scales, and perceptual test.	Structural equation modeling of scale item responses and dimensional measures.
