# Supplementary material for: Quantum evidence of nonlocal consciousness during clinical death
Source: Innovation (Camb). 2026 Mar 25;7(9):101355. doi: 10.1016/j.xinn.2026.101355 (PMC13556340; doi:10.1016/j.xinn.2026.101355)
Supplement: Supplementary file 2 [file mmc2.pdf]

ARTICLE | Biomedical Engineering

OPEN ACCESS

Quantum evidence of nonlocal consciousness during clinical death

Álex Escolà-Gascón,[1,*] Kenneth Drinkwater,[2] Andrew Denovan,[3] Neil Dagnall,[2] and Julián Benito-León[4,5,6,7,*]

*Correspondence: aescola@icade.comillas.edu (Á.E.-G.); julianbe@ucm.es (J.B.-L.)

Received: October 16, 2025; Accepted: March 20, 2026; Published Online: March 25, 2026; https://doi.org/10.1016/j.xinn.2026.101355

© 2026 The Author(s). Published by Elsevier Inc. on behalf of Youth Innovation Co., Ltd. This is an open access article under the CC BY license (http://creativecommons.org/licenses/by/4.0/).

www.the-innovation.org

GRAPHICAL ABSTRACT

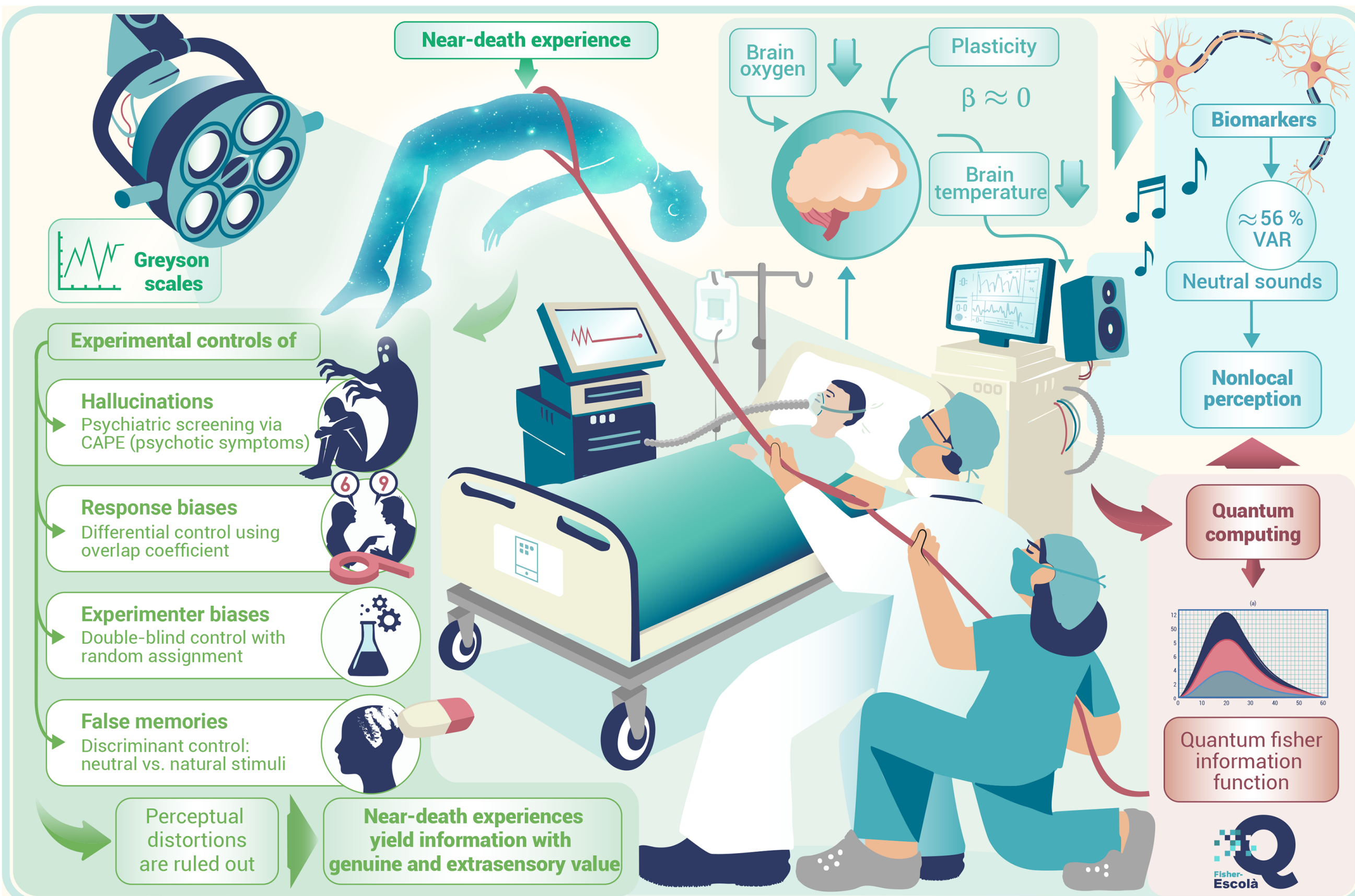

PUBLIC SUMMARY

- Fisher-Escolà *Q* coefficient quantifies entanglement-behavior effects with validated thresholds.
- Entanglement increases accurate recall; non-entangled controls remain at chance performance.
- Expectation bias eliminated: entangled groups shared recall distributions, overlap coefficient 0.843.
- Nonlocal perception predicted 32% of near-death experience variance, independent of psychotic symptoms.
- False-memory and hallucination controls isolate entanglement-driven recall in resuscitated patients.
- We deliver clinical guidance on monitoring thresholds, communication, sedation, and post-resuscitation care.

Quantum evidence of nonlocal consciousness during clinical death

Álex Escolà-Gascón,[1,*] Kenneth Drinkwater,[2] Andrew Denovan,[3] Neil Dagnall,[2] and Julián Benito-León[4,5,6,7,*]
[1]Department of Quantitative Methods and Statistics, Comillas Pontifical University, 28015 Madrid, Spain
[2]Faculty of Health, Psychology and Social Care, Manchester Metropolitan University, Manchester M15 6GX, UK
[3]School of Psychology, Liverpool John Moores University, Liverpool L3 5UX, UK
[4]Department of Neurology, 12 de Octubre University Hospital, 28041 Madrid, Spain
[5]Instituto de Investigación Sanitaria Hospital 12 de Octubre (imas12), 28041 Madrid, Spain
[6]Network Center for Biomedical Research in Neurodegenerative Diseases (CIBERNED), 28029 Madrid, Spain
[7]Department of Medicine, Faculty of Medicine, Complutense University of Madrid, 28040 Madrid, Spain
*Correspondence: aescola@icade.comillas.edu (Á.E.-G.); julianbe@ucm.es (J.B.-L.)
Received: October 16, 2025; Accepted: March 20, 2026; Published Online: March 25, 2026; https://doi.org/10.1016/j.xinn.2026.101355
© 2026 The Author(s). Published by Elsevier Inc. on behalf of Youth Innovation Co., Ltd. This is an open access article under the CC BY license (http://creativecommons.org/licenses/by/4.0/).
Citation: Escolà-Gascón Á., Drinkwater K., Denovan A., et al., (2026). Quantum evidence of nonlocal consciousness during clinical death. The Innovation **7(9)**, 101355.

If consciousness can operate under quantum principles, then the boundaries between life, death, and cognition are far more permeable than current science allows. In the first large-scale, randomized, double-blind, multicenter trial to test this possibility, conducted across 13 hospitals in the UK and Spain, we investigated whether the human mind can access information during clinical death—but only when exposed to auditory stimuli governed by quantum entanglement. The entangled stimulation circuit was implemented and synchronized with the 127-qubit IBM Brisbane quantum supercomputer and delivered during in-hospital cardiopulmonary arrest (CA), beginning 120 s after arrest onset. Quantum entanglement was verified through statistically significant violations of Mermin inequalities. During resuscitation, we measured neurophysiological biomarkers including cerebral oxygenation via near-infrared spectroscopy (NIRS), brain temperature, and blood pH, lactate, and serum concentrations of brain-derived neurotrophic factor (BDNF). After reanimation, 142 survivors completed a forced-choice recall test, near-death experience (NDE) scales, and other clinical control assessments. False memories, hallucinations, and confounding biases were successfully controlled. Notably, recall lucidity increased momentarily as NIRS values dropped, and NDEs positively correlated with neuroplasticity during CA. Biomarker models explained up to 56.8% of the variance in recall. These findings compel a radical rethinking of clinical death: consciousness may persist—quantum bound, detectable, and not yet defeated.

INTRODUCTION

According to Greyson,[1] approximately 11% to 22% of studies investigating near-death experiences (NDEs) report negative or distressing phenomenological content. NDEs are subjective perceptions that arise in moments preceding death, during clinical death, deep coma, or critical conditions in which a patient undergoes cardiopulmonary arrest (CA) and requires resuscitation.[2–4] Upon surviving these life-threatening episodes, individuals frequently describe vivid, lucid experiences perceived during states in which, physiologically speaking, the necessary conditions for consciousness or information processing should not have been present.[5] One critical question has persistently captured scientific attention: if the physiological substrates for conscious experience were ostensibly absent, how can such individuals report coherent experiences at all?[6]

For decades, researchers have sought to explain NDEs within various theoretical frameworks. Explanations range from plausible psychological mechanisms—such as imagination,[7] lucid dreaming,[8,9] or even false memories and perceptual illusions to more speculative and controversial hypotheses,[10] including the proposition that NDEs may reflect nonlocal aspects of consciousness or evidence of conscious survival beyond physical death.[11,12] Numerous definitions and conceptualizations of NDEs have been proposed,[13] yet even among leading experts in the field, no unified consensus has emerged regarding their nature.[14] This conceptual heterogeneity presents a significant challenge—not because the proposed explanations lack merit but because scientific inquiry is inherently limited by methodological constraints that are ill-equipped to capture the full empirical and phenomenological complexity of the event.[1]

In this study, we focus on a specific subset of NDEs referred to as experiences of death (DEs)—episodes that occur under empirical conditions that, by current neuroscientific standards, should preclude the emergence of vivid subjective awareness.[15] According to Parnia et al.,[15] an NDE should only be considered genuine or anomalous when it arises in the context of verifiable, life-threatening events or states of dying consciousness. While this criterion has faced criticism for being reductive and excluding the rich qualitative dimensions of survivor reports,[14,16] it remains, for now, an operational and clinically functional benchmark for conducting research under conditions of maximum scientific rigor. Abandoning this criterion in favor of a broader definition—one that includes all anomalous perceptions near the end of life, such as terminal lucidity—would dilute the epistemic significance of NDEs.[17] Such inclusivity would risk assimilating NDEs into known or hypothesized biological and neurological mechanisms that, though perhaps still undiscovered, would situate them within the broader class of perceptual anomalies—distortions, illusions, and visions—that frequently occur in the terminal phase of illness.[18,19] In such cases, any experimental study would suffer a critical loss of internal validity, undermining the ability to determine whether NDEs constitute truly anomalous or scientifically extraordinary events.[20,21]

This study contends that, despite its limitations, the criterion proposed by Parnia et al.[15] provides a rigorous and much-needed framework that positions NDEs where they arguably belong: as phenomena that challenge prevailing biophysical and medical models of conscious survival, for example, interpreting such NDEs as forms of conscious awareness disconnected from the body and advocating for their classification as a subtype of out-of-body experience (hereafter OBE).[5,22] Although OBEs represent a broader and less controversial domain of inquiry than the survival hypothesis,[20] they offer a valuable conceptual and descriptive scaffold for advancing scientific understanding of NDEs.[23] Despite the controversies sparked by the work of Parnia et al.,[15,24] even their critics—such as Martial et al.[25]—have underscored the essential importance of methodological and conceptual rigor in this domain. This position aligns with the fundamental values of responsible scientific inquiry.

Are NDEs explainable as hallucinations or false memories?

Some researchers have argued that NDEs may be anticipated based on the formation of false memories.[26,27] Yet, such findings do not conclusively establish that NDEs are fabricated or illusory,[28] nor does the inability to verify these experiences against objective events necessarily imply that they are entirely confabulated[29]; a similar ambiguity arises when NDEs are interpreted as hallucinations. In a comprehensive medical review, Parnia[30] concluded that NDEs constitute a distinct domain of investigation, separate from end-of-life hallucinations. Along the same lines, Kellehear,[31] in a systematic clinical review, noted that while hallucinations are not uncommon during the final stages of terminal illness, their diagnosis remains fraught with ambiguity and methodological imprecision.

A number of studies have sought to clarify the phenomenological distinctions between end-of-life hallucinations and NDEs. From this body of research, three key differentiators emerge:

(1) Hallucinations typically lack internal coherence and present disorganized, often irrational content. In contrast, NDEs tend to follow a structured, meaningful narrative, as reported by experiencers themselves, and are consistently described as lucid.[25]
(2) Hallucinations generally do not evoke themes of transcendence, spirituality, or existential meaning, nor do they necessarily reflect an

individual's belief system. NDEs, on the other hand, often have profound spiritual implications, even for children.[32]

(3) Hallucinations rarely induce lasting psychological transformation. They do not appear to significantly alter cognitive frameworks, values, or personality traits. NDEs, by contrast, often result in profound and paradoxically positive changes in individuals' lives, despite their implausibility.[33,34]

To these phenomenological criteria, one can add the operational definition advanced by Parnia et al.,[15] which stipulates that an NDE must occur under verifiable physiological conditions of a critical, life-threatening episode with loss of consciousness, in which no conscious experience should be neurologically feasible.[35] This criterion has gained traction in recent empirical research and has been independently supported by phenomenological investigations,[36] lending it a degree of scientific reliability.

Beyond phenomenology, quantitative and statistical studies also point to significant divergences between hallucinations and anomalous experiences such as NDEs. For instance, Escolà-Gascón and Rusiñol[37] have shown that these phenomena exhibit distinct psychometric profiles. Although such findings have received limited attention within mainstream NDE research, they support the hypothesis that NDEs may reflect a form of anomalous cognition fundamentally different from classically pathological hallucinations. Of course, the absence of a definitive explanation does not, by itself, confirm that consciousness operates independently of brain function. Nonetheless, these empirical insights provide legitimate scientific grounds for continued inquiry—especially into explanatory frameworks that extend beyond the limits of orthodox neuroscience.[38,39]

Moreover, studies of individuals who experienced CA and were successfully resuscitated have yielded compelling empirical evidence suggesting that NDEs cannot be reduced to mere illusions, perceptual errors, or cognitive distortions. Instead, they appear to reflect a distinct class of experience that resists explanation by conventional physiological models.[24,40]

The materialist paradigm in the study of NDEs

Hess[41] proposes that scientific attempts to explain NDEs can be broadly classified into two main categories. The first comprises neuroscientific theories that aim to identify the physiological mechanisms underlying these experiences. These accounts, firmly situated within the field of neuroscience, are fundamentally ontological, aiming to determine the primary source of NDEs. For example, Nelson[42] suggested that NDEs may result from cerebral responses to abrupt and maladaptive fluctuations in blood flow, especially during transitions between different levels of conscious activation. Similarly, Peinkhofer et al.[5] hypothesized that NDEs could emerge from hormonal fluctuations that perturb the temporal regions of the brain, leading to unconscious pseudo-hallucinations that patients later recall as vivid experiences. Other models, direct and indirect neuronal frameworks, further expand the scope of this explanatory approach.[43] While diverse in formulation, these theories all operate within the boundaries of the scientific materialist paradigm.

The second category comprises psychological theories that frame NDEs as affective or cognitive responses to the perceived threat of death. These perspectives do not aim to resolve the ontological question of origin; instead, they explore the psychological functions and long-term consequences of NDEs in patients' lives. Here, the focus shifts from causality to meaning—how these experiences shape human behavior, learning, and cultural engagement.[44]

Applied to the study of consciousness, scientific materialism imposes an epistemic framework that often renders NDEs elusive or resistant to explanation. As Greyson[45] has argued, the positivist and mechanistic assumptions underpinning mainstream scientific inquiry struggle to accommodate phenomena that appear to defy reductionist modeling. This challenge has prompted a growing number of scholars to call for expanded theoretical paradigms—ones capable of integrating the subjective richness of conscious experience without relinquishing empirical rigor.[46] NDEs are frequently cited as exemplars of phenomena that remain inaccessible to existing scientific methods and narratives.[28]

In fact, as early as the mid-1990s, philosopher and neuroscientist David Chalmers[47] articulated what has become known as the "hard problem of consciousness"—the challenge of explaining how and why subjective experiences arise from physical processes. Although initially unrelated to NDEs, the conceptual obstacles Chalmers identified are directly relevant to this field of inquiry.[48] Like the hard problem itself, NDEs force us to confront the limitations of reductive explanations for conscious phenomena.

We do not presume to offer a definitive solution to the apparent shortcomings of materialist science. However, we argue that the path forward does not lie in rejecting materialism but in evolving it. Rather than opposing the scientific paradigm, it may be more productive to extend it—to develop empirically grounded frameworks that allow NDEs to be studied under controlled or quasi-experimental conditions. Such an approach would mirror the way science investigates other complex, internally experienced phenomena—such as appetite, pain, intelligence, obsession, or humor—whose authenticity is not in question despite their subjective nature. In what follows, we propose a revised but scientifically coherent model for exploring the anomalous, extraordinary, and presently unexplained content of NDEs.

Nonlocality theories in the context of NDEs

In quantum physics, nonlocality refers to the capacity of certain subatomic phenomena to transcend conventional spatiotemporal constraints—they are not bound to a fixed location in space or time.[49] This concept has inspired a number of theoretical models, proposing that an as-yet-undiscovered aspect of conscious experience may operate according to nonlocal principles.[50] It is essential, however, to distinguish the hypothesis of nonlocal consciousness from the ontological claim that consciousness is a fundamental or pre-physical substrate of reality.[51] While the two perspectives may intersect, the present analysis adheres strictly to the notion of nonlocality as defined by von Lucadou,[52] emphasizing its operational implications rather than its metaphysical assumptions.

Within this paradigm, von Lucadou[53] introduced the pragmatic information model to account for anomalous experiences such as premonitory dreams and precognition,[54] and Grinberg-Zylberbaum[55,56] contributed the syntergic theory of consciousness, while Penrose—a Nobel laureate in physics—advanced the orchestrated objective reduction model, attributing conscious awareness to quantum processes occurring within neural microtubules.[57] More recently, Walach et al.[58] provided empirical evidence supporting the plausibility of nonlocal dynamics in consciousness, a line of inquiry further explored in the work of Kauffman and Radin.[59] At a more applied level, Escolà-Gascón[60] proposed the nonlocal plasticity theory (NPT), a model positing that implicit learning may occur through translocalized morphological changes in the synaptic architecture of the central nervous system. These developments offer compelling grounds for exploring NDEs through the lens of nonlocal consciousness. However, despite their theoretical promise, nonlocality-based frameworks remain epistemologically inconclusive: they do not, at present, allow us to determine whether NDEs reflect the actual continuation of consciousness beyond physical death or whether they merely reveal unknown cognitive mechanisms by which the brain accesses information through non-classical channels.[61]

The principal strength of nonlocality theories lies not in their capacity to define the outer limits of scientific possibility but in their formalization of information matrices (IMs). These are stochastic mathematical constructs that conceptualize consciousness as a dynamic interplay between environmental information fields, as proposed by Walach et al.,[58] and phenomenological structures, as articulated by Grinberg-Zylberbaum.[55,56] Within this framework, the brain is conceived as a system capable of "scanning" information of such matrices, much as it processes sensory stimuli through traditional perceptual modalities (touch, sight, smell, taste, and hearing).

Despite this theoretical coherence, two major scientific challenges remain unresolved:

(1) There is still a lack of mechanistic understanding of how the brain accesses nonlocal information. This epistemic gap was underscored by Utts (1996)[62] in her technical reports for the US government, which, although focused on anomalous cognition, highlighted unresolved questions that are equally pertinent to the study of NDEs.

(2) The origin and nature of the information encoded within IMs remain undefined. Several speculative models have attempted to address this, including Grinberg-Zylberbaum et al.'s lattice matrix[63] and Escolà-Gascón's[60] framework of translocalized neuroplasticity.

For nonlocality theories to be fully integrated into the scientific mainstream, the informational content of IMs must ultimately be shown to correlate with measurable dimensions of objective reality. This requirement of empirical verifiability remains the central unresolved challenge—and the most significant test for the future of nonlocal models in the scientific study of consciousness and NDEs.

Objectives and hypotheses of the present study on NDEs

This study aimed to investigate the empirical and statistical relationships among several key variables: (1) a set of biomarkers routinely recorded in the field of intensive care medicine, as detailed in the materials and methods section, (2) standardized psychometric scales assessing the phenomenology and transformative impact of NDEs, (3) binary outcomes generated by an IBM-certified quantum random event generator (QREG), and (4) two independent experimental tests designed to assess potential nonlocal features of NDE-related processes.

Our research framework rests on two complementary pillars. The first is theoretical, grounded in the nonlocality models previously outlined in the nonlocality theories in the context of NDEs. The second is empirical: irrespective of philosophical positions or conceptual critiques, scientific inquiry relies fundamentally on replicability as a benchmark for validating the robustness and generalizability of observed phenomena. In this context, it is worth underscoring that the findings reported by Parnia et al.[24] have yet to be independently replicated and must therefore be regarded, at best, as anomalous results awaiting external confirmation. As an autonomous research group, we sought to rigorously test those findings using a new cohort of patients who experienced CA and survived. Accordingly, the convergence of theoretical innovation and the imperative of replication underpins both the rationale and the relevance of the present study. Within this framework, we formulated three central hypotheses.

(1) A component of conscious experience operates in a nonlocal manner, such that it may interact with the quantum collapses generated by QREGs (hypothesis of consciousness transcending the biological confines of the body) and may access environmental or informational stimuli without recourse to the established sensory modalities recognized by conventional neuroscience (hypothesis of nonlocal access to information).
(2) At least one biomarker measured during CA will show a significant correlation with the collapse events recorded by the QREG, supporting an ontological hypothesis that links material physiological markers to the possible emergence of nonlocal conscious states. In other words, if consciousness does in fact interact with the entangled quantum states underlying QREG outputs, this interaction may become biologically traceable through specific organic variables during the near-death episode. This hypothesis challenges the presumed dichotomy between material embodiment and nonlocal cognitive phenomena.
(3) At least one biomarker measured during CA will correlate with the phenomenological dimensions of the NDE, as assessed by psychometric instruments. While structurally analogous to the previous hypothesis, this one substitutes quantum measurements with subjective experiential scales, thereby advancing a sociocultural hypothesis that seeks to bridge the gap between first-person experience and its underlying physiological substrates.

Grounding our hypotheses in the quantum framework

The ontological validity of NDEs as genuine perceptual events should not be assessed solely through cultural or medical lenses. As scientists, we must subject to empirical testing the possibility of anomalous connections between the dying states that occur during CAs and the brain's hypothetical capacity to detect and store unique environmental stimuli during such critical periods of prolonged CA—periods that, from the standpoint of Newtonian physics, should be entirely devoid of cognitive or perceptual function. This points to the possibility of nonlocal and, hypothetically, quantum processes. Such speculation about the relationship between quantum phenomena and the boundaries of conscious experience—such as NDEs—should not remain merely philosophical. Scientific inquiry must rigorously examine this possibility and provide evidence to support its validity, should it prove to be real.

Our approach, grounded in the ontological principles of the NPT, posits that the environmental configuration of stimuli and their exposure are what cognitively enable the brain—possibly through plastic mechanisms—to generate credible signals or reactions in response to information from external reality. Escolà-Gascón[60] demonstrated experimentally that this possibility can occur through systematic variation via a form of quantum-like implicit learning. As in Escolà-Gascón's[60] design, the stimuli in each trial in this study were assumed to adopt quantum states that, through controlled manipulation, could form latent pattern structures during collapses, ultimately influencing the stimuli's environmental exposure. Such a structure could be realized using entangled qubit states, leveraging IBM's operational quantum computers within the noisy intermediate-scale quantum (NISQ) paradigm.

If a quantum-level process influences stimulus exposure, which in turn produces perceptual changes during NDEs, then empirically testing this hypothesis requires conceptualizing each trial as a qubit capable of triggering the presentation of a specific auditory stimulus. The crucial point is that, *a priori*, the occurrence of such exposure is entirely uncertain or indeterminate—this indeterminacy being what may suggest the involvement of quantum processes in conscious experiences, including NDEs. Consequently, our hypothesis also accepts as plausible the idea that the indeterminacy regarding whether a stimulus is presented in each trial may be analyzed according to the mathematical rules of quantum physics.

On the one hand, in the absence of any manipulation of the qubit states, the collapses resulting from superposition (modeled using Hadamard gates) should be completely random. This implies that the configuration of stimuli across each patient's trial sequence should also be random (the design of the experimental procedures employed in this study is detailed in the materials and methods section). If this assumption holds, we would expect no quantum effect (i.e., no latent patterns across the set of collapses).

On the other hand, if we were to manipulate the qubit states to generate entanglement among them deliberately, we would thereby introduce nonlocal correlations into the density matrix of those states. Depending on the nature of the entanglement, it is conceivable that an anomalous transfer of influence could occur, linking the qubit entangled states to the binary collapses. In this case, although the binary collapses might appear random from a classical macroscopic standpoint, at the quantum level (which we manipulate via qubit states), such randomness should not exist because the nonlocal correlations would be significantly greater than zero within the density matrix. Thus, at the quantum level, there would be latent patterning in the qubit states that could meaningfully influence the collapses and, by extension, the exposure of auditory stimuli. We refer to this idea as the hypothetical theory of latent patterns, developed mathematically by Escolà-Gascón and Benito-León.[64] This study aims to test whether quantum latent patterns might underlie the type of entanglement used to present auditory stimuli during the prolonged CAs of patients undergoing NDEs. This theory will be further elaborated in the discussion section, in light of the findings obtained.

This reasoning provides a framework for bridging the deterministic universe of macroscopic reality with the indeterministic origins of the quantum world. If each qubit is treated as a trial in which binary collapse determines whether a stimulus is presented (event-value 1) or not (event-value 0), and we manipulate the uncertainty in the states (within the density matrix, via the backend of the quantum processor executing the circuit) by inducing nonlocal correlations and entanglement—without any hidden variable being able to classically explain the latent matrix structure (i.e., violating Bell's inequality)—then these quantum structures could influence how each qubit collapses. Such an effect-transfer mechanism might statistically predict perceptual phenomena during NDEs—phenomena that would remain undetectable or non-replicable under classical paradigms but could, under quantum conditions, contribute explained variance suitable for empirical and realist testing within classical frameworks.

MATERIALS AND METHODS

Patient sample characteristics

Estimated sample size. To ensure sufficient statistical power in our analyses, we conducted a preliminary sample-size estimation based on the study design. The power threshold was set at 99%, assuming moderate standardized effect sizes (d = 0.5). We used the phi coefficient and the estimation equations proposed by Escolà-Gascón.[65] Our calculations indicated that a minimum of 45–50 cases per group was needed to detect

significant effects. Since the study included three groups, the total sample size ranged from 135 to 145 participants. While this number might seem small, it entailed enormous logistical challenges.

Inclusion-exclusion criteria. If the incidence of CAs in European hospitals fluctuates between 11% and 17%,[66] then it would be necessary to monitor and screen thousands of patients at high risk of experiencing a CA. This cardiovascular risk factor was the primary indicator through which it became potentially feasible to recruit patients who had survived a CA and also reported an NDE. To this incidence rate, we must add the average survival rate for such patients in intensive care units, which ranges between 25% and 30%.[67] From this already limited proportion, we must also account for the proportion of studies reporting negative NDEs among survivors, which reaches up to 22%.[1]

In addition, to accurately present patient follow-up statistics, the following inclusion and exclusion criteria that shaped the sampling process were also considered:

(1) Patients had to present a high risk of suffering a CA.
(2) Patients had to be adults aged 45 to 65 years.
(3) Patients had to be hospitalized under close monitoring in an intensive care unit (e.g., postoperative care, resuscitation units, and observation units), with a minimum hospital stay of 3 days.
(4) The CA episodes had to be prolonged, lasting 120–360 s, exposing patients to low blood oxygen levels, physiological sensory loss, and the need for assisted artificial respiration.
(5) Patients had to survive the CAs they experienced. Although some might present neurotrophic damage, such impairments could not incapacitate their judgment or hinder their ability to complete memory-based evaluation tasks and the psychometric scales used in the study (see the subsection instruments).
(6) During their high-risk phase, patients had to consent to participate in the study voluntarily. They agreed to provide non-personal, non-identifiable medical data on five physiological recordings collected during their hospitalization and to complete a series of cognitive assessments and perception-related questionnaires.
(7) Patients with a history of neurodegenerative diseases or terminal illnesses or those in palliative care were excluded from the study due to the high dropout or mortality rates typically associated with such conditions, whether from incapacity or death.

Given those criteria and the logistical demands of the study, collaboration with 13 medical institutions was required (all of which were operated by Catholic religious orders). These institutions collectively monitored between 15,000 and 15,300 patients at high risk of CA. Among them, 2,418 patients suffered a CA and received cardiopulmonary resuscitation (CPR). Unfortunately, only 677 survived. These survivors were asked whether they had perceived any form of lucid experience at the time of the CA, including NDEs and OBEs.

Following this extensive screening process, a final cohort of 142 patients was assembled: 47 in experimental group A, 49 in control group B, and 46 in control group C (details regarding the conditions of each group are provided in the subsection methodological and mathematical procedures). Random assignment was carried out on a 1:1:1 basis prior to the patient entering CA. The research team had a maximum window of 110 s to complete randomization, implement the appropriate quantum system, and apply auditory stimuli according to the group to which the patient had been assigned. If a patient was resuscitated within 120 s, the experimental procedure was canceled. Likewise, if the patient died during the resuscitation attempt or entered an irreversible coma, the experiment was either not conducted or, if already performed, the corresponding data were discarded on ethical grounds (storing unnecessary information and overburdening the data registry with disposable records could have contributed to increased fatigue among the medical staff who generously collaborated in this project on a voluntary basis).

Double-blinded sampling controls. The patients who agreed to participate in this study were recruited from 13 hospitals and clinics in Spain and the United Kingdom—all of which chose to collaborate anonymously. The inclusion of medical centers from different countries rendered this a multicenter study, thereby increasing the internal validity of its procedures.

At the beginning of the sampling process, patients at high risk of cardiovascular disease were identified. Each of these individuals was informed about the nature of the study, and consent was requested to access non-identifiable medical data regarding specific biological markers analyzed during potential CAs—see the subsection instruments. Patients were then randomly assigned to one of three groups using the Python Qiskit library: group A (experimental), group B, or group C.

All participants received the same information and were unaware of their group assignment. Likewise, the scientists who authored this article were blind to each patient's group assignment. These assignments were managed confidentially by a research assistant technician, who maintained absolute discretion in allocating patients to each group.

In each hospital, a research assistant was responsible for managing and ensuring the implementation of the double-blind procedure for both physicians and patients. The investigators trained these assistants, but there was no exchange of results among them until after the interventions and data collection were completed. These controls enabled us to prevent experimenter bias by implementing double-masked procedures, a standard practice in experimental research design.

Sample description. The final sample of patients with CA analyzed for this study consisted of 142 participants who voluntarily and anonymously agreed to participate. Of these, 109 were male, and 33 were female, with ages ranging from 49 to 65 years (mean = 58.41; standard deviation = 3.1). All patients met the inclusion and exclusion criteria outlined in the subsection inclusion-exclusion criteria. 71 patients were recruited from institutions in the United Kingdom and 71 from institutions in Spain. The international sampling process was supported by the international research consultancy CIE Consulting.

Quantum circuit design and applications

Quantum circuit configurations with and without entanglement. Two 10-qubit circuits were designed for this study. The first, "CM1," applied a Hadamard gate to each qubit prior to measurement. This configuration ensured a superposition state between 0 and 1, resulting in maximal uncertainty and completely random collapse sequences. The second circuit, "EM1," applied a Hadamard gate only to the first qubit, while the remaining qubits were entangled through various configurations of CNOT gates. These settings ensured the presence of nonlocal correlations that entangled the qubit states. In addition, EM1 included randomly weighted rotation gates to introduce controlled noise, preventing invariant collapse patterns (e.g., rows consisting entirely of 0s or 1s). Circuit EM1 was used for both experimental group A and control group B, while circuit CM1 was assigned exclusively to control group C. The purpose of this configuration was to test whether structuring the experimental trials of the cognitive auditory task—based on nature sounds and neutral melodies (see the subsection methodological and mathematical procedures for further details)—using entangled qubits could produce a transfer effect capable of predicting a portion of the variance and structure in the participants' response matrices.

The detailed graphical representations of both circuits (CM1 and EM1) are provided in the supplemental information accompanying this publication. This supplemental information contains the complete schematics and technical specifications required to fully support and complement the explanation developed in this subsection.

Each trial functioned analogously to a qubit, capable of existing in superposed and entangled states with respect to two possible outcomes: option 1 involved projecting the auditory stimulus during the CA for 20 s, while collapse to 0 involved withholding the stimulus during the same event. In every trial, a 0 or 1 collapse was generated to determine whether the auditory stimulus would be presented.

If the entanglement of qubits were capable of transferring an effect—namely, modulating collapse outcomes in the absence of hidden variables in the density matrix that could explain or predict such variation—then we would be observing a form of cognitive and perceptual functioning with quantum underpinnings. This is not an idle claim. Individual experience and perception are known to be shaped by environmental signals and stimuli, even when these are subtle. If entanglement influenced the collapse outcomes in the EM1 circuit, then, within the logic of our experimental and empirical design, it would also affect the configuration of stimulus presentation (since a collapse to 1 triggered exposure, whereas a collapse to 0 did not). This experimental paradigm, which integrates classical and quantum approaches, had never been attempted before. It may represent a highly valuable mathematical and methodological innovation, not only for advancing basic scientific understanding of consciousness but also for enhancing medical studies aimed at predicting the survival of patients resuscitated after prolonged CA.

Mathematical demonstrations. The initial state of the 10 qubits in the EM1 circuit is defined in Equation 1:

$$|0\rangle^{\otimes 10} = |0\rangle_1 \otimes |0\rangle_2 \otimes \cdots \otimes |0\rangle_{10}. \quad \text{(Equation 1)}$$

The application of the Hadamard gate to the first qubit (q_0) produces a superposition state that maximizes uncertainty (Equation 2):

$$H_1|0\rangle_1 = \frac{1}{\sqrt{2}}\left(|0\rangle_1 + |1\rangle_1\right). \quad \text{(Equation 2)}$$

The application of CNOT gates, which generate nonlocal correlations, between qubits 1 and 2 through 10 can be formulated as presented in Equation 3. This equation represents the Greenberger-Horne-Zeilinger state (GHZ):

$$|\mathrm{GHZ}_{10}\rangle = \frac{1}{\sqrt{2}}\left(|0\rangle^{\otimes 10} + |1\rangle^{\otimes 10}\right). \quad \text{(Equation 3)}$$

As shown in the circuit, two types of local random rotations, denoted as $R_y(\theta)$ and $Rz(\Phi)$, were applied. These rotations introduced deviations to diversify the collapse outcomes while preserving the quantum coherence of the entanglement (see Equation 4):

$$R_y(\theta) = \begin{pmatrix} \cos\frac{\theta}{2} & -\sin\frac{\theta}{2} \\ \sin\frac{\theta}{2} & \cos\frac{\theta}{2} \end{pmatrix}, R_z(\Phi) = \begin{pmatrix} e^{-i\Phi/2} & 0 \\ 0 & e^{-i\Phi/2} \end{pmatrix}, \quad \text{(Equation 4)}$$

and the final prepared state was as shown in Equation 5:

$$|\psi\rangle = \bigotimes_{j=1}^{10} \left[R_z^{(j)}(\Phi_j) R_y^{(j)}(\theta_j) \right] |\mathrm{GHZ}_{10}\rangle. \quad \text{(Equation 5)}$$

To preserve nonlocal correlations and obtain a density matrix that violates Mermin's inequality,[68,69] the angle range was set as $\theta_j, \Phi_j \in [0, \pi/20]$, ensuring small rotations. Local collapse generation, in contrast, involved angles spanning the full range $[0, 2\pi]$.

In two-qubit circuits, the violation of the Clauser-Horne-Shimony-Holt (CHSH) inequality serves as the standard criterion for ruling out hidden variables in the density matrix.[70,71] However, in more complex systems involving three or more qubits—each with two possible states—the mathematical framework must account for higher-order interactions that extend beyond simple qubit pairs.

In this study, the verification of Mermin's inequality violation (denoted as M_{10}) follows the formulation presented in Equation 6:

$$M_{10} = \frac{1}{2} \left[\bigotimes_{j=1}^{10} \left(\sigma_x^{(j)} + i\sigma_y^{(j)} \right) + \bigotimes_{j=1}^{10} \left(\sigma_x^{(j)} - i\sigma_y^{(j)} \right) \right]. \quad \text{(Equation 6)}$$

Both σ_x and σ_y are the Pauli matrices defined in Equations 7 and 8:

$$\sigma_x = \begin{pmatrix} 0 & 1 \\ 1 & 0 \end{pmatrix} \quad \text{(Equation 7)}$$

and

$$\sigma_y = \begin{pmatrix} 0 & -i \\ i & 0 \end{pmatrix}. \quad \text{(Equation 8)}$$

From this point onward, the Mermin expected value was computed using Equation 9:

$$\langle M_{10} \rangle = \mathrm{Tr}(M_{10}\rho), \quad \text{(Equation 9)}$$

where $\rho = |\psi\rangle\langle\psi|$ is the density matrix of the prepared state (see Equation 5). For an ideal GHZ state (in the absence of significant perturbations), the expected result is $2^{10-1} = 512$. This value surpasses the classical limit, confirming a violation of Mermin's inequality. Our density matrix for this circuit has dimension 210 × 210, yielding up to 1,048,575 independent quantum correlations; therefore, we cannot present the full computation for all of them. To specifically identify correlations associated with nonlocality, we analyze only those coefficients in which a non-identity Pauli operator acts on every qubit. For instance, an N-partite correlation would be computed following Equation 10:

$$C_{\mathrm{Nonlocal}} = \mathrm{Tr}\left[\rho\left(\sigma_{i_1} \otimes \sigma_{i_2} \otimes \cdots \otimes \sigma_{i_N}\right)\right], \quad \text{(Equation 10)}$$

with $ik \in \{1,2,3\}$ for all k.

At this stage, it is important to recall that a violation of Mermin's inequality implies the absence of hidden variables within a classical (or non-quantum) framework capable of explaining the structure of the density matrix values. This validation confirmed that the EM1 circuit contained qubits in entangled states prior to collapse.

Methodological and mathematical procedures

The study's design and procedures, conducted in accordance with the CONSORT guidelines for randomized trials, are classified as experimental and randomized. The protocol employed a double-blind methodology and incorporated between-group comparisons alongside structural equation modeling (SEM) to assess the hypothesized relationships. This study included three groups: group A was the experimental group and groups B and C served as control conditions to prevent biases related to experimenter influence, false memories, and response conformity. At this point, we highlight that the CONSORT flow diagram illustrating the allocation of cases to the study's experimental conditions, along with its description, is available as supplemental information in the online version of the article.

Characteristics and conditions of experimental group A. If a patient, unfortunately, suffered a prolonged CA lasting at least 2 min and was successfully resuscitated, then, from 120 s into the period of unconsciousness, up to 5 different environmental sounds representing natural phenomena were projected auditorily in 20-s intervals. These included stimuli with distinct wave functions and acoustic features, such as crashing ocean waves, light rain, wind, fire, and birdsong. To prevent sound projection from interfering with medical resuscitation protocols, auditory stimuli were delivered via a speaker connected to a laptop, which was brought into the operating room after stimulus selection (within 0–90 s of CA onset). Depending on the system configuration and technical specifications, this laptop was sometimes used to remotely synchronize with the IBM Brisbane supercomputer, streamlining stimulus selection and delivery. In other cases, a laptop preloaded with potential stimuli was prepared in advance, and the selected sounds were played based on the choices made by another technically equipped computer synchronized with IBM Brisbane.

If the patient survived the CA and reported having had some form of NDE, in addition to the measurement of medical markers (see the subsection biomarkers) and the administration of the near-death experience scale (NDE-S) by Greyson[3] (see the subsection Greyson scales for NDEs), the patient was also required to complete a brief cognitive experimental test. In this test, the patient listened to a sequence of 10 natural sounds, five of which had been previously projected during the CA. At the same time, the remainder served as distractors for recall discrimination. Patients were asked to indicate which sounds they believed they had perceived during their CA. Each correct identification—where the patient's memory matched the actual projected auditory stimulus—was scored as "1." Incorrect responses were recorded as "0." Since the micro-test consisted of 10 perceptual-experimental trials, the maximum possible score was 10, and the minimum was 0.

For clarity: if NDEs were merely subjective phenomena lacking any meaningful connection to external reality at the moment of death, then patients' correct recall scores should not exceed chance-level expectations based on statistical uncertainty. However, if NDEs involve some form of perceptual-physical linkage with the environment—even during periods when the brain is deprived of oxygen and should be unable to generate subliminal perceptual states—then it would be reasonable to expect some level of recall. Moreover, this memory performance could be analyzed in relation to the subsequently reported NDE. This was the central rationale of our study.

Characteristics and conditions of control groups B and C. It is essential to highlight the distinction between control groups B and C to understand and justify the methodological controls implemented.

Control group B patients received the same instructions as those in experimental group A. This means that group B also employed the EM1 quantum circuit, utilizing qubit entanglement to configure the stimuli. The key difference from group A was that the auditory stimuli in group B consisted entirely of new melodies, purposefully created for this research. To generate these unique and emotionally neutral melodies, we used artificial intelligence tools, specifically Magenta Studio and Pixabay.

The rationale for using melodies as auditory stimuli was to control for biases that can lead to false memories. If a survivor reported having had an NDE and was asked about the presence or absence of specific stimuli during the period of that experience, we needed to ensure that memory distortions did not influence their memory discrimination. This is a particularly challenging issue in the context of NDEs. However, the methodology of our design enabled us to draw a comparison that helped distinguish between patients whose responses were affected by false memories and those whose responses were not. Whereas a natural environmental sound might be mistakenly identified by a patient as something perceived during the NDE, when in fact it could have originated from unrelated prior experiences, substituting those natural sounds with entirely novel melodies (that had never been published or played before the CA) created a type of stimulus that could not easily be confounded through false memory mechanisms.

Patients randomly assigned to the control group C were exposed to the same neutral melodies as those used in group B during their CA, with one critical difference: in group C, stimulus exposure was not based on qubit entanglement. Instead, it was entirely random, lacking any latent patterning. This control condition allowed us to isolate more variables and potential confounding factors, making it the most restrictive baseline for bias control.

By comparing the responses of patients in group B with those in group A, we assessed whether unconscious response biases influenced patients' recall. Such biases—encompassing a variety of unconscious tendencies—can emerge when patients, without realizing it, attempt to tailor their responses to align with what they perceive as the researchers' expectations.[72] To ensure that this type of bias did not predict the responses of patients in group A, we examined the degree of pattern similarity between the response structures of groups A and B. If significant discrepancies emerged between these patterns, this would provide evidence of unconscious response bias.

Instruments

Biomarkers. The biomarkers measured in this study were selected based on prior research suggesting possible associations between these physiological parameters and NDEs. Table 1 summarizes the instrumental measurements used, including cerebral oxygenation (measured using near-infrared spectroscopy [NIRS]), blood lactate

Table 1. Description of the biomarker devices and their characteristics

Biomarkers	What is measured?	Type of device	Procedure	Metrics
Cerebral oxygenation (NIRS)	oxygen saturation in the cerebral cortex reflects real-time perfusion and neuronal metabolism; it is based on the differential absorption of oxygenated hemoglobin (HbO_2) and deoxygenated hemoglobin (HbR)	INVOS 5100C (from Medtronic)	an NIRS sensor is placed on the patient's forehead, emitting infrared light to measure differential absorption in the cerebral cortex; this enables estimation of regional oxygen saturation (rSO_2)	10%–20% cerebral oxygen saturation (rSO_2) due to the cessation of cerebral perfusion
Blood lactate	blood lactate concentration, reflecting anaerobic metabolism in response to tissue hypoxia; higher hypoxia leads to increased lactate production as a byproduct of anaerobic cellular metabolism	Siemens RAPIDPoint 500	arterial blood is drawn and analyzed using a blood gas analyzer (point-of-care testing [POCT]) that measures lactate via enzyme immunoassay or spectrophotometry	>10 mmol/L lactate
Blood pH	the acid-base balance in arterial blood; a decrease in pH indicates metabolic acidosis due to lactic acid accumulation under severe hypoxia	Siemens RAPIDLab	arterial blood is analyzed with a blood gas analyzer to measure the hydrogen ion (H^+) concentration, which indicates the patient's acid-base status	7.0–7.1, which are logarithmic metrics
Brain temperature	the brain's core temperature reflects metabolic activity and perfusion status; a rapid drop in temperature after cardiopulmonary arrest indicates neuronal metabolic shutdown, while abnormal stability or an increase could indicate residual metabolic activity	Philips BrainCool IQool System	brain temperature is measured using intracranial temperature probes (for invasive monitoring) or tympanic thermometers (for non-invasive approximation)	34.5°C–35.4°C
Brain-derived neurotrophic factor (BDNF) or plasticity predisposition	levels of interaction between proteins and the TrkB tyrosine kinase, which mediates the activation and regulation of neuroplasticity	brain-derived neurotrophic factor (BDNF) Rapid ELISA Kit (CE marked), provided by Avantor	ELISA (enzyme-linked immunosorbent assay)	1–15 ng/mL

concentration, blood pH levels, and brain temperature, which provide insights into metabolic and perfusion dynamics under extreme hypoxic conditions.

Data collection was conducted using hospital-grade equipment, including the INVOS 5100C (Medtronic) for cerebral oxygenation, the Siemens RAPIDPoint 500 and RAPIDLab analyzers for blood lactate and pH measurements, and the Philips BrainCool IQool System for brain temperature monitoring. As a biomarker of particular interest, we included measurements of brain-derived neurotrophic factor (BDNF), which assesses proteins that interact with the tyrosine kinase B (TrkB) receptor—key regulators of neuronal synapses.[73] BDNF has been linked to core processes involved in memory and perception,[74] both of which may play a role in NDEs. We sought to explore whether the brain's plastic mechanisms could act as mediators in extending conscious experience beyond the currently known biological limits. Given that BDNF does not directly measure the plastic changes occurring in the nervous system but rather indicates a predisposition for morphological change in neural networks, we aimed to assess whether individuals with higher levels of BDNF-TrkB protein-receptor interactions might report more vivid NDEs with greater attribution of meaning.

Likewise, when tested with both natural and neutral auditory stimuli, it would not be implausible that individuals with elevated BDNF levels might retain their perceptual faculties for longer periods—even under prolonged and critical CA conditions—allowing them to register and later recall environmental information.

All the information in Table 1 and the measurements specified therein did not interfere with the resuscitation process, as all instruments were part of the standard monitoring procedures in the collaborating hospitals, which participated anonymously in this research.

Greyson scales for NDEs. Greyson[3] was the first to develop a multidimensional scale designed to measure the extent to which an NDE constitutes a genuinely transcendent phenomenon and a challenge to scientific understanding. To help distinguish between NDEs that may be mere hallucinations or extraordinary perceptions without meaningful personal impact, the Greyson NDES includes four dimensions encompassing a total of 16 items. Each item offers three response options, scored 0, 1, or 2, depending on the perceived intensity of the experience described. In this scoring system, 0 represents the lowest intensity, while 2 indicates the highest.

The four dimensions assessed by the NDES are cognitive, affective, paranormal, and transcendental. The specific items for each dimension were originally published by Greyson[3] and have since been psychometrically re-evaluated by independent researchers, who confirmed the scale's validity and reliability.[13,75,76] In our sample, the internal consistency coefficients exceeded 0.8 for each of the four subscales and for the total score, supporting the reliability of the scores for research purposes.

The NDES construct emphasizes that the more meaningful and coherent the patient's perceived experience is, the greater the likelihood that it is not a mere hallucination but rather a psychological resource capable of driving learning or personal transformation. Thus, the NDES does not aim to assess the ontological origin of NDEs—neither affirming nor denying any supernatural value—but instead emphasizes the psychological utility these experiences may hold for individuals and their impact on perception and worldview.[1]

Table 2 presents the reliability coefficients for the NDES and the experimental protocol described in the following section, based on our patient sample.

Community Assessment Psychic Experiences-42 (control). Stefanis et al.[77] developed the Community Assessment of Psychic Experiences-42 (CAPE-42), designed to assess symptomatology across the psychosis spectrum through three dimensions that classify symptom types: (1) the negative dimension of psychosis (focused on social and cognitive difficulties), (2) the positive dimension of psychosis (focused on perceptual disturbances and delusional ideation), and (3) the depressive dimension (focused on emotional difficulties).

In this study, we were interested in determining whether NDEs—often confused with hallucinations—could be formally distinguished from the perceptual alterations observed in psychotic conditions. Our objective was not to conduct a clinical psychiatric evaluation of such symptoms; rather, we aimed to use only the 17 items from the positive dimension as a statistical control technique to explore potential associations with NDEs. Therefore, the remaining items from the CAPE-42 were excluded and not administered to the patients in this study.

The CAPE-42 items consist of direct questions in which participants indicate, on a four-point Likert scale, how frequently they experience the specified symptoms, ranging from 0 ("never") to 3 ("almost always"). For the positive dimension, the total possible score ranged from 0 to 51.

In this study, the original English version of the CAPE-42[77] was used for the UK sample, and the Spanish adaptation by Ros-Morente et al.,[78] later validated by Fonseca-Pedrero et al.,[79] was used for the Spanish sample. Reliability coefficients for the positive dimension of the CAPE-42 are presented in Table 2.

The results presented in Table 2 indicate that all three reliability coefficients exceeded 0.7, suggesting that the measurements were sufficiently reliable for research purposes. Moreover, NDEs exhibited negative correlations with the positive dimension of the CAPE-42 scale, suggesting that although they may superficially resemble hallucinations, NDEs are complex experiences that fall outside the psychotic spectrum. This statistical evidence supports the conclusion that the NDEs recorded in our study were not hallucinations and did not share the symptomatic features typically associated with the perceptual disturbances observed in psychosis.

Table 2. Reliability coefficients of the measurements obtained from the psychometric scales used in this study within our sample of patients

	Correlations among scales	α	ω	GLB	Average inter-item correlation
NDE cognitive dimension	−0.543[a]	0.748	0.752	0.8	0.43
NDE affective dimension	−0.454[a]	0.712	0.727	0.731	0.466
NDE paranormal dimension	−0.292[a]	0.756	0.759	0.812	0.435
NDE spirituality dimension	−0.287[a]	0.723	0.725	0.767	0.394
NDE Greyson scale total scores	−0.536[a]	0.88	0.883	0.927	0.255
Positive dimension of the CAPE-42 scores (psychotic-like experiences)	–	0.925	0.925	0.967	0.425

α, Cronbach's alpha coefficient; ω, McDonald's omega coefficient; GLB, greatest lower bound.
[a]$p < 0.01$.

The Fisher-Escolà *Q* coefficient

Escolà-Gascón[80] proposed and demonstrated the predictive efficacy of a new coefficient—the quantum multilinear integrated coefficient, denoted as Q—intended to measure the proportion of explained variance relative to entanglement in a bipartite system when applied to the configuration of stimulus contingencies within a nonlocal learning design. In Equation 11, we present the coefficient Q:

$$Q = V_k \cdot \left(1 + \beta \cdot C_q \cdot S\right), \quad \text{(Equation 11)}$$

where V_k is the explained variance obtained from the latent factors of matrix responses, β is the weight that enables the modulation of quantum effects, C_q is the quantum concurrence,[81] and S is the value of the Bell inequality obtained from nonlocal correlations according to the CHSH criteria.

In its bipartite version (for systems of two quantum objects or qubits), Q is a functional and effectively captures explained variance by combining classical latent patterns with the percentage of quantum information derived from entanglement. However, our current system involves 10 qubits, which requires Q to be adapted, since the value S, based on the CHSH criterion, focuses only on pairs of nonlocal correlations. Furthermore, the accuracy and stability of S are not comparable to the Mermin value, which, when generating varied combinations with multiple qubits, loses metrological consistency. Although this does not necessarily affect the decision about whether the qubit states are entangled, it could negatively impact the calculation of the Q statistic. A solution to this issue is found in the improved version of the Q statistic: the Fisher-Escolà Q ($Q_{\text{Fisher-Escolà}}$) for multipartite systems, as is the case here. All mathematical and statistical information is presented in Escolà-Gascón and Benito-León,[64] but the formulas used in this study are shown in Equations 12 and 13:

$$Q_{\text{Fisher-Escolà}} = V_k\left(1 + \beta C_q\left(4 \cdot \frac{\int_{\lambda_{\min}}^{\lambda_{\max}} \frac{(\partial_\theta \lambda)^2}{\lambda} d\lambda}{\max f(\lambda)}\right)\right) \Rightarrow V_k\left[1 + \beta C_q \cdot \frac{4\int_{\lambda_{\min}}^{\lambda_{\max}} \frac{(\partial_\theta \lambda)^2}{\lambda} d\lambda}{\max f(\lambda)}\right] \quad \text{(Equation 12)}$$

or

$$Q_{\text{Fisher-Escolà}} = V_k\left(1 + \beta C_q (4 \cdot I_Q)\right), \quad \text{(Equation 13)}$$

where λ is the eigenvalue of the ρ density matrix and θ represents the evolution parameter of the quantum system. The $Q_{\text{Fisher-Escolà}}$ is based on the quantum Fisher information (QFI), originally developed by Fisher,[82] and is applied to the system's density matrix in the presence of entangled states.[83] QFI measures the extent to which information (variability) flows within a quantum system and is accessible for understanding its effects. A quantum system with extremely low QFI may exhibit maximal coherence and pure entanglement; however, this would also imply that the system's states are insensitive to variation, rendering its information hermetic or analytically inaccessible. Conversely, a quantum system with high QFI will experience decreased coherence among its states, making it more sensitive to contextual changes (decoherence) and producing local variations that increase the system's information accessibility.

The problem with using QFI is that excessive system sensitivity makes it more vulnerable to decoherence, potentially nullifying its quantum properties and thereby truncating or distorting its characteristics. Similarly, excessively low sensitivity would hinder analytic access to the system's information (although its quantum properties would remain intact). Understanding the logic of QFI implies that the goal should be to obtain, ideally, moderate sensitivity values—values that preserve the quantum properties of entanglement while simultaneously allowing a level of variability or accessible information compatible with classical statistical analysis.

What Escolà-Gascón and Benito-León[64] proposed was not to use the QFI as a mathematical function per se but rather a specific version of its integral. The integral of the QFI yields the cumulative probability density function, which quantifies the degree of certainty (or probability) that a quantum system stores a given amount of accessible information. While the QFI reflects a system's sensitivity to changes, its integral estimates how much accessible information the system accumulates as a function of that sensitivity. The particular version of the integral proposed by Escolà-Gascón and Benito-León[64] is shown in Equation 12, where the integral is divided by the maximum value of its function, centered on the eigenvectors (λ), and thus represented as max$f(\lambda)$. This adjustment normalizes I_Q values to the range [0,1].

For the explained variance $Q_{\text{Fisher-Escolà}}$ to increase and not remain static, the following conditions must be met: $\beta > 0$, $C_Q > 0.5$, and $I_Q \approx 0.5$. The purpose of using the $Q_{\text{Fisher-Escolà}}$ was to capture the proportion of variance attributable to the quantum effects of entanglement. Therefore, in addition to satisfying these mathematical conditions, it was also necessary for $Q_{\text{Fisher-Escolà}} > 0$. To test this final hypothesis, the theoretical Q distribution of Fisher-Escolà must be used, as previously indicated. The $Q_{\text{Fisher-Escolà}}$ distribution follows the beta(a, b, loc, $scale$) distribution. Type I and II errors were evaluated in an independent study using this new distribution across 10^5 Monte Carlo estimates, and error rates did not exceed 5% for either type of hypothesis test.[64] Furthermore, as the significance level decreased, both error types also declined, reaching values as low as 0.1%.

This information is relevant because the theoretical $Q_{\text{Fisher-Escolà}}$ distribution—still relatively unknown—demonstrated equivalent statistical power and error risk to that of the classical Fisher's F distribution. The critical values for $Q_{\text{Fisher-Escolà}}$ were 0.392, 0.429, and 0.459 for significance levels (α) of 0.01, 0.001, and 0.0001, respectively. Thus, our null hypothesis stated that $Q_{\text{Fisher-Escolà}} \leq Q_\alpha$. Given that a one-tailed right-sided test was employed, if $Q_{\text{Fisher-Escolà}} > Q_\alpha$, this implied that $P(Q_\alpha \geq Q_{\text{Fisher-Escolà}}) < \alpha$. Failure to reject the null hypothesis would indicate that the variation in $Q_{\text{Fisher-Escolà}}$ was not statistically significant and could be attributed to random perturbations—thus supporting the conclusion that no quantum effects were involved in the patients' responses.

Ethical compliance statement

The research protocols and study design received favorable evaluations from the institutional ethics committees of the universities affiliated with the authors and were further reviewed and approved by the two corresponding authors. All participants were fully informed and provided their voluntary consent prior to inclusion. Moreover, we certify that all procedures conformed to the ethical standards outlined in the latest revision of the Declaration of Helsinki (2013), thereby safeguarding the dignity, rights, and well-being of all individuals involved.

RESULTS

Because the methodological information is extensive and detailed, we have provided a PDF with summaries and specifications as supplemental information to help readers more easily follow all the methodological steps in our design. We recommend accessing it through the online version of this work.

Quantum properties

Using Python v.3.9.21, the Mermin inequality was computed for the density matrices generated by the EM1 circuits assigned to groups A and B. By analyzing this inequality—alongside measurements of multipartite quantum

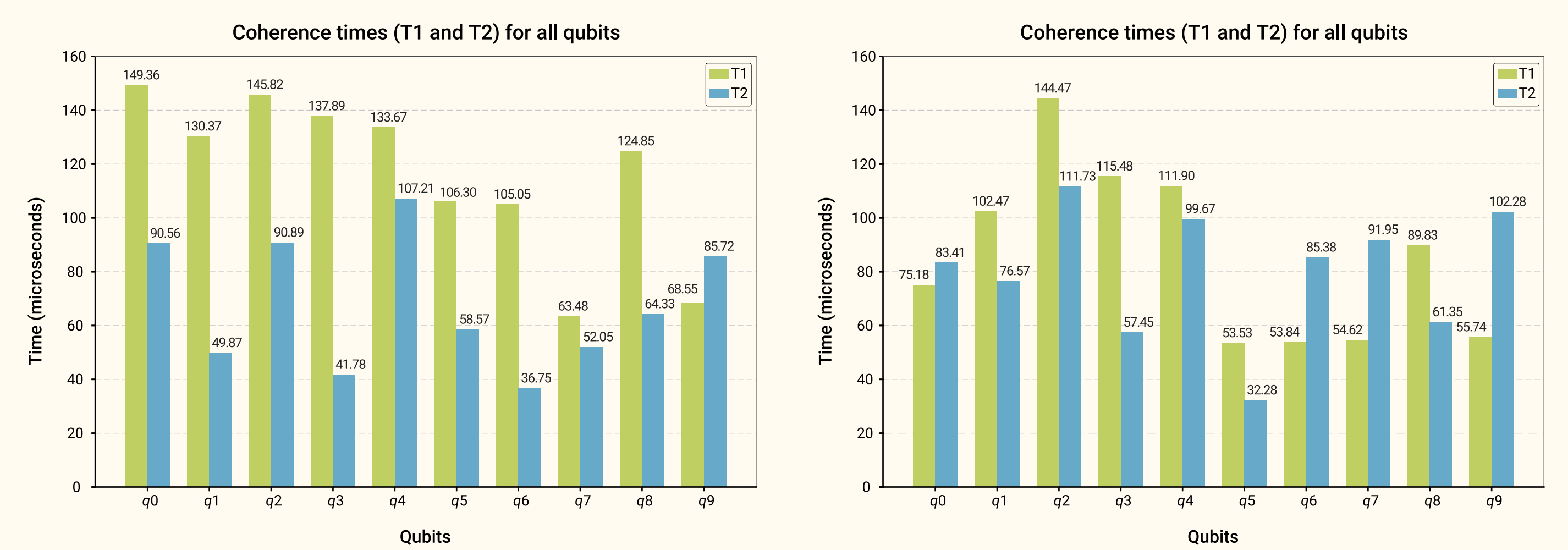

Figure 1. Descriptive plots showing the average coherence times for each qubit Bar charts displaying the coherence times of qubits in response to circuit execution (the first chart corresponds to group A, while the second represents group B). The low microsecond values indicate optimal circuit processing, ensuring that these delays did not compromise the quantum accuracy of the entangled states.

concurrence and von Neumann entropy—we were able to verify whether the qubit states were quantum entangled and whether this entanglement influenced the binary measurement/collapse outcomes. The expected value was obtained using Equation 9. In our case, the value was near the threshold of 23, with 512 representing the theoretical upper limit, as previously justified in the subsection quantum circuit design and applications. The closer the Mermin operator's value approaches 512, the greater the degree of entanglement. Conversely, if the value falls below 23, there would be mathematical grounds to assert that the qubit states are not entangled.

The results for the EM1 circuit showed Mermin-GHZ values of 368.7117 and 347.6603 for groups A and B, respectively, indicating that no hidden variables could account for the observed qubit-state correlations. The values of quantum concurrence—an index that specifically measures the degree of entanglement—were 0.9997 for the density matrix of group A and 0.9994 for group B. To assess the purity of the entanglement and determine whether the qubit states were pure or mixed, we also analyzed the von Neumann entropy. In this design, the closer the entropy value is to 1, the less pure the entanglement, indicating the presence of noise within the entangled states. The entropy values obtained were 0.0025 for group A and 0.0054 for group B. Given the low values observed (close to 0), we can confidently assert that the entanglement was predominantly pure, though not perfectly so (with total purity approximating 0.00001). We also automated the computation of QFI integrals according to the I_Q logic. These values were essential because they enabled us to apply the $Q_{\text{Fisher-Escolà}}$ statistic in conjunction with the weighting coefficient β, which represents the explained variance. This variance was calculated via the omega-squared (ω^2) coefficient derived from the coefficient derived from the one-way ANOVA conducted in the experiment (see the subsection experimental contrast: analysis of variance). The I_Q values were 0.5001 and 0.5002 for groups A and B, respectively.

Finally, Figure 1 presents the average reaction times of the qubits used in the IBM Brisbane quantum processor. Reaction time, or coherence time, is measured in microseconds and serves as a control variable to ensure that the IBM quantum processor did not introduce temporal delays (quantified as an error) that could bias the measurement/collapse process and compromise the internal validity of the experiment.

The results shown in Figure 1 indicate that there were no significant delays in qubit reaction times. Accordingly, we obtained acceptable coherence times without affecting the density matrices or the measurement/collapse processes. Thus, the binary collapses observed in groups A and B under the EM1 circuit were driven by qubits entangled at a relatively high level of purity.

Experimental contrast: analysis of variance

Table 3 summarizes analyses comparing means and variances across groups A, B, and C, along with the corresponding descriptive statistics. These analyses examined the effects of biomarkers, verified the presence of entanglement effects (under the assumption of a transition from quantum-entangled states to individual responses in perceptual tasks), and included controls for false memory biases.

It is important to clarify that these biomarker comparisons are used to characterize the physiological context of CA and to confirm baseline comparability between groups rather than to infer differences in clinical outcomes. Because all participants met the same inclusion criteria and completed follow-up, the study was not designed to test between-group differences in endpoints such as survival to discharge or neurological outcomes. Consistent with this design, most biomarkers showed no statistically significant differences across groups (e.g., NIRS, blood lactate, and plasticity predisposition), supporting broadly similar physiological conditions during CA.

Two biomarkers reached conventional statistical significance: arterial blood pH ($p = 0.002$; $\omega^2 = 0.070$) and brain temperature ($p = 0.015$; $\omega^2 = 0.045$). However, statistical significance does not necessarily imply clinical relevance. The absolute between-group differences were small (mean pH: 6.95–6.98, maximum difference: 0.03 units; mean brain temperature: 34.80°C–34.97°C, maximum difference: 0.17°C), and all values remained within the severe ranges expected during prolonged CA and states of clinical or near-clinical death. Clinically, differences of this magnitude are unlikely to be actionable or to reflect meaningfully different degrees of systemic or cerebral insult between groups.

The statistical separation for pH is largely driven by very low within-group dispersion (standard deviation range: 0.033–0.034) and by the logarithmic scaling of the pH metric; a 0.03-unit shift reflects a small proportional change in hydrogen ion concentration due to the logarithmic scaling of pH and does not move patients into a different clinically defined acid-base category. Likewise, the observed temperature difference lies within the monitored brain-temperature range defined in the materials and methods section and is unlikely to be clinically meaningful. Given the number of biomarkers assessed, isolated p values close to the nominal significance threshold should also be interpreted cautiously as potential type I findings. Accordingly, we interpret the pH and brain temperature effects as statistically detectable but clinically trivial baseline differences unlikely to confound the study's primary outcomes. Future replications could prespecify clinically meaningful thresholds, report confidence intervals for between-group differences, and model biomarkers longitudinally during and after CA to better distinguish statistical from clinical significance.

However, the variable anomalous cognitions did yield significant differences, with standard deviations that were not low when considered against the variation expected by chance under the classical probability paradigm: $\sqrt{½ \times ½ \times 10} = 1.58$. In fact, from a statistical perspective, when comparing the observed standard deviations with the expected estimate under chance, we find that the differences are not attributable to random variation. This implies that if the mean scores for this variable differed, such differences could not be attributed solely to chance. This conclusion is supported by the F value, which was relatively high (15.198), indicating that the means—6.68 for group A,

Table 3. Analysis of variance and descriptive statistics for all dependent variables in the study

Variables	Group	*N*	Mean	SD	*F*	*p* value	Partial η^2	ω^2
**Biomarkers (A = natural, B = neutral, and C = neutral without entanglement)**								
NIRS	A	47	14.87	3.012	1.398	0.251	0.020	0.006
	B	49	15.00	2.858	–	–	–	–
	C	46	14.07	2.932	–	–	–	–
Blood lactate	A	47	16	2.638	0.307	0.736	0.004	∼0
	B	49	16.31	3.595	–	–	–	–
	C	46	16.52	3.365	–	–	–	–
Blood pH	A	47	6.98	0.034	6.358**	0.002	0.080	0.070
	B	49	6.96	0.033	–	–	–	–
	C	46	6.95	0.034	–	–	–	–
Brain temperature	A	47	34.97	0.307	4.347*	0.015	0.059	0.045
	B	49	34.80	0.286	–	–	–	–
	C	46	34.83	0.291	–	–	–	–
Plasticity	A	47	8.34	2.838	2.834	0.062	0.039	0.025
	B	49	7.51	3.228	–	–	–	–
	C	46	6.85	3.003	–	–	–	–
**Greyson's near-death experiences scale (A = natural, B = neutral, and C = neutral without entanglement)**								
NDE cognitive	A	47	4.23	2.189	1.339	0.266	0.019	0.005
	B	49	3.82	2.078	–	–	–	–
	C	46	3.54	1.87	–	–	–	–
Affective	A	47	4.15	2	0.346	0.708	0.005	∼0
	B	49	3.88	1.889	–	–	–	–
	C	46	3.83	2.174	–	–	–	–
Paranormal	A	47	4.11	1.97	0.8	0.452	0.011	∼0
	B	49	3.69	1.906	–	–	–	–
	C	46	3.65	1.9	–	–	–	–
Spirituality	A	47	3.66	1.992	2.115	0.124	0.030	0.015
	B	49	4.37	1.922	–	–	–	–
	C	46	4.33	1.7	–	–	–	–
Greyson scale total scores	A	47	16.30	6.032	0.322	0.725	0.005	∼0
	B	49	15.76	5.491	–	–	–	–
	C	46	15.35	5.662	–	–	–	–
**Sound identification and positive dimension of the CAPE-42 scores (A = natural, B = neutral, and C = neutral without entanglement)**								
Sounds identification[a] (anomalous cognition)	A	47	6.68	1.353	15.198***	<0.001	0.180	0.170
	B	49	6.00	1.581	–	–	–	–
	C	46	4.94	1.665	–	–	–	–
Positive dimension of the CAPE-42 scores (psychotic-like experiences)	A	47	15.30	8.556	0.129	0.879	0.002	∼0
	B	49	16.31	10.302	–	–	–	–
	C	46	15.96	10.587	–	–	–	–

*$p < 0.05$, **$p < 0.01$, and ***$p < 0.001$. SD, standard deviation.
[a]The experimental task included ten auditory stimuli, of which only five had actually been played during the resuscitation phase following CA. These five served as target stimuli presented under conditions of either quantum entanglement or classical randomness. The remaining five were false (non-projected) sounds, included to assess participants' ability to discriminate true stimuli from false ones and to control for false memories using a 50% counterbalancing procedure. As such, participant scores in Table 3 range from 0 to 10, corresponding to the total number of stimuli presented during the recognition test. This design addresses potential memory biases commonly observed in near-death experiences, as discussed by Martial et al.[29]

www.the-innovation.org

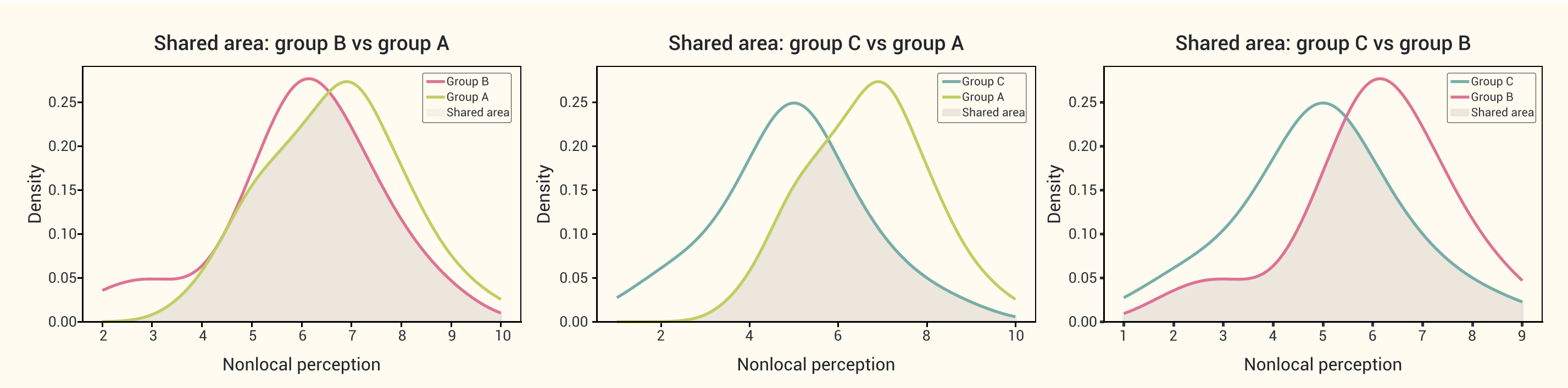

Figure 2. A statistical analysis of the area under the curve shared by two or more distributions was conducted using the overlapping coefficient An overlapping coefficient (OVL) value above 0.7 was considered indicative of substantial overlap or near equivalence between distributions. The most critical group comparison in this analysis was the plot contrasting the distributions of groups (A and B).

6.00 for group B, and 4.94 for group C—showed systematic, non-random variation. These variations were further quantified using the proportion of explained variance, which ranged from 17% to 18%. Therefore, for this variable, there was indeed anomalous variation attributable to qubit entanglement, and this finding should be highlighted as both significant and suggestive.

At this point, however, it is worth considering whether the score of 6.00 (group B) or 6.68 (group A) might reflect biases arising from false memories. Due to our experimental design, we tested this possibility using the Student's t distribution (note that this was a one-tailed right-sided test). We applied a Bonferroni correction to the p value. Our analysis revealed that the difference between scores of 6 and 6.68 was not statistically significant ($p \approx 1$), which supports the conclusion that the 6.00 score—corresponding to neutral stimuli projected with quantum entanglement—was not due to false memory errors.

To detect false memories or such biases, group B would have had to score significantly *higher* than group A. This clarification, although it adds length to the paper, is essential for understanding the logic of our analysis of false memory control. The control variable measuring psychotic symptoms also showed no significant results, indicating that the exposure to entangled stimuli, if it produced any effect, was not associated with increases in clinically relevant psychotic symptoms. Taken together with the linear correlation reported in Table 2, these findings reinforce our conclusion that NDEs are not hallucinatory, nor do they imply psychotic risk for the individuals reporting them.

To investigate whether there was any response bias related to expectation adjustment, we analyzed the distributions of correct responses on the nonlocal perception test by computing the areas under the curve (AUCs) shared between these distributions across groups A, B, and C. The method used to calculate these integrals was the overlapping coefficient (OVL), a statistically validated technique for estimating shared AUCs between groups.[84]

It is important to recall that, to rule out expectation bias, the distributions of groups A and B should yield a high OVL value. Following the threshold proposed by Clemons and Bradley,[85] this value should ideally exceed 0.7. Figure 2 shows the shaded overlapping AUCs corresponding to the nonlocal perception test distributions for groups A, B, and C.

The results indicated an OVL of 0.843 between groups A and B, 0.584 between A and C, and 0.683 between B and C. With the exception of the AUC shared between groups A and B, all other OVL values were below the 0.7 threshold, suggesting that there were notable discrepancies in the distribution of correct responses for group C.

These findings are consistent with the results obtained from the one-way ANOVA analyses discussed earlier and support the conclusion that this type of response bias did not influence any participant responses. Consequently, the mean accuracy scores of groups A and B can be considered equivalent, and the value for group B did not exceed that of group A—indicating that neither false memories nor expectation bias likely played a role in the observed outcomes.

SEM and multilevel analysis

In the multiple regression analyses we conducted, we aimed to address two important questions. The first was to determine the extent to which biomarkers could predict total Greyson scale scores for NDEs. Identifying potential neural correlates and comparing them with previous findings in this area is crucial for a deeper understanding of the boundaries of conscious experience under extreme physiological conditions. In other words, if NDEs were merely hallucinations, false memories, or response biases, then the predictive patterns between biomarkers and these three control variables (hallucinations and biases) should not differ structurally or statistically from those observed with genuine NDEs (where a genuine NDE is defined as involving some aspect of consciousness persisting beyond the biological limits of near-death states).

The second analysis explored the relationship between correct responses in the sounds identification task and scores on psychotic symptomatology. As shown in Table 2, there were negative correlations between Greyson scale scores and CAPE scores, suggesting that the phenomenological features of psychotic hallucinations differ from those of NDEs. Nonetheless, we were prepared to evaluate this issue with greater statistical rigor and in finer detail.

To properly test the hypothesis that NDEs are genuine, we would expect similarly negative correlations between CAPE scores and performance on the nonlocal perception task. A near-zero (non-significant) correlation between CAPE scores and anomalous cognitions would also support the dissociation between hallucinations and nonlocal perception in the context of NDEs. However, if a positive correlation were found between CAPE and nonlocal perception, we would then have empirical and statistical grounds to support claims from other studies suggesting that NDEs may be ontologically treated as hallucinations or perceptual distortions within the psychosis spectrum.

For the first set of analyses, we fitted ordinary least squares (OLS) regression models, using biomarkers as exogenous variables and nonlocal perception accuracy (anomalous cognitions) as the endogenous variable. For the analyses exploring the relationships among NDEs, CAPE, and nonlocal perception, we employed SEM to provide more stringent control for CAPE's effects on NDEs, as measured by the Greyson scale.

Linear regression analysis. The biomarkers utilized in this study are enumerated in Table 1. Our first step was to present the correlation matrix between biomarkers, the dimensions of the Greyson scale, and nonlocal perception scores. As shown in Table 4, this provides a broad overview of the role each NDE dimension may have played.

The correlation matrix revealed sufficiently significant associations to support the development of multiple group-specific regression models. Our analyses focused on predicting the endogenous variables: total NDEs and nonlocal perception. Across all model versions presented below, we employed ordinary least-squares regression with forward stepwise selection, as this approach is well suited for identifying the most optimal and parsimonious predictive models.

On the one hand, for the total NDE scores, we found the following results. For experimental group A, $\beta_{NIRS} = -0.9580$ (standard error = 0.248, $t = -3.856$, $p < 0.01$) and $\beta_{Blood\ pH} = -45.600$ (standard error = 21.792, $t = -2.092$, $p < 0.05$). The adjusted R^2 for the A model was 0.451 (45.1%, $F = 20.68$, $p < 0.01$). For control group B, $\beta_{NIRS} = -0.9539$ (standard error = 0.254, $t = -3.749$, $p < 0.01$), $\beta_{Blood\ lactate} = 0.612$ (standard error = 0.246, $t = 2.494$, $p < 0.05$), and $\beta_{Blood\ pH} = -51.316$ (standard error = 24.390, $t = -2.104$, $p < 0.05$). The adjusted R^2 for the B model was 0.493 (49.3%, $F = 15.92$, $p < 0.01$). For control group C, $\beta_{Blood\ pH} = -57.504$ (standard error = 19.753,

Table 4. Linear Pearson correlation matrix between biomarkers and the dimensions of the Greyson NDE scale, including the variable of nonlocal perception

	NIRS	Blood pH	Blood lactate	Brain temperature	Plasticity predisposition
**Group A (*N* = 47)**					
NDE cognitive	−0.474**	−0.171	0.482**	−0.559**	0.326*
NDE affective	−0.596**	−0.555**	0.062	−0.272	−0.082
NDE paranormal	−0.247	−0.331*	0.142	−0.351*	−0.046
NDE spirituality	−0.576**	−0.452**	0.252	−0.428**	0.102
NDE total scores	−0.652**	−0.502**	0.306*	−0.526**	0.094
Nonlocal perception	−0.501**	−0.067	0.554**	−0.628**	0.453**
**Group B (*N* = 49)**					
NDE cognitive	−0.337*	−0.411**	0.292*	−0.154	0.111
NDE affective	−0.625**	−0.601**	0.073	−0.486**	−0.184
NDE paranormal	−0.444**	−0.218	−0.023	−0.054	−0.228
NDE spirituality	−0.440**	−0.322*	−0.200	−0.228	−0.440**
NDE total scores	−0.651**	−0.551**	0.058	−0.324*	−0.254
Nonlocal perception	−0.401**	−0.307*	0.208	−0.341*	−0.012
**Group C (*N* = 46)**					
NDE cognitive	−0.375*	−0.371*	−0.336*	−0.384**	0.332
NDE affective	−0.260	−0.416**	−0.094	−0.482**	0.214
NDE paranormal	−0.219	−0.418**	−0.319*	−0.276	0.294*
NDE spirituality	−0.267*	−0.253	0.024	−0.269	0.184
NDE total scores	−0.378**	−0.499**	−0.247	−0.485**	0.346*
Nonlocal perception	−0.138	−0.359*	−0.101	0.110	0.011

*$p < 0.05$ and **$p < 0.01$.

$t = -2.911$, $p < 0.01$), $\beta_{\text{Brain temperature}} = -7.9158$ (standard error = 2.229, $t = -3.552$, $p < 0.01$), and $\beta_{\text{NIRS}} = -0.4759$ (standard error = 0.229, $t = -2.080$, $p < 0.05$). The adjusted R^2 for the C model was 0.427 (42.7%, $F = 12.18$, $p < 0.01$).

On the other hand, regarding nonlocal perception, we obtained the following predictions, some of which reached statistical significance or showed marginal effects. For experimental group A, $\beta_{\text{Brain temperature}} = -1.326$ (standard error = 0.593, $t = -2.236$, $p < 0.05$), $\beta_{\text{Blood pH}} = 0.124$ (standard error = 0.069, $t = 1.792$, $p = 0.080$), $\beta_{\text{NIRS}} = -0.175$ (standard error = 0.066, $t = -2.643$, $p < 0.05$), and $\beta_{\text{Plasticity}} = 0.114$ (standard error = 0.064, $t = 1.765$, $p = 0.085$). The adjusted R^2 for the A model was 0.568 (56.8%, $F = 13.08$, $p < 0.01$). For control group B, $\beta_{\text{NIRS}} = -0.198$ (standard error = 0.082, $t = -2.412$, $p < 0.05$) and $\beta_{\text{Blood lactate}} = 0.105$ (standard error = 0.058, $t = 1.809$, $p = 0.077$). The adjusted R^2 for the B model was 0.196 (19.6%, $F = 4.910$, $p < 0.05$). For control group C, $\beta_{\text{Blood pH}} = -18.908$ (standard error = 6.865, $t = -2.754$, $p < 0.01$), and $\beta_{\text{NIRS}} = -0.4759$ (standard error = 0.229, $t = -2.080$, $p < 0.05$). The adjusted R^2 for the C model was 0.121 (12.1%, $F = 4.101$, $p < 0.05$). Figure 3 presents the scatterplots and fitted lines for these exogenous variables (only the significant ones) in both models.

The regression analyses indicated that NIRS, brain temperature, blood pH, and blood lactate biomarkers, together, accounted for 49.3% of the variance in perceived NDEs. For nonlocal perception, the maximum explained variance reached 56.8%. These findings suggest that both NDEs and nonlocal perception may have biological correlates linked to the dying process, offering insight into why such experiences emerge in some patients but not in others. However, despite these significant associations, more than 40% of the variance in both NDEs and nonlocal perception remains unexplained. Importantly, because we controlled for response biases, false memories, and hallucinations, we can reasonably exclude these factors as primary contributors to the remaining unexplained variance. This point is further examined in the discussion within a clinical framework.

SEM. In our structural equation models, parameter estimation was performed using mean- and variance-adjusted weighted least squares (WLSMV), with weights derived from the trace of the error matrix. This approach enabled us to optimize the correlations between ordinal item-level variables and their corresponding constructs while accounting for measurement error in the latent factors—an element typically overlooked in classical regression models. Accordingly, this step strengthened statistical control. These results are illustrated in Figure 4.

The fit indices for the predictive model of NDEs in Figure 4, accounting for positive psychotic symptoms and nonlocal perception, were $\chi^2 = 766.564$ (degrees of freedom = 551, $p < 0.001$), root-mean-square error of approximation (RMSEA) = 0.058 (lower bound = 0.049 and upper bound = 0.066, acceptance threshold $\leq$ 0.08), comparative fit index (CFI) = 0.951 (acceptance threshold > 0.9), Tucker-Lewis index = 0.950 (acceptance threshold > 0.9), and weighted root-mean-square residual (WRMR) = 1,080 (acceptance threshold $\leq$ 1 with middle and high loadings). For the WRMR indicator, we tested the null hypothesis that the observed WRMR value equals the theoretical threshold of 1 to determine whether the 0.08 difference could be attributed to chance. If 1.080 is not significantly different from 1, this would provide evidence that the observed WRMR aligns with the maximum threshold for acceptable model fit. We conducted a one-tailed right-sided test using the Z distribution, setting 1 as the theoretical threshold and 1.080 as the observed value (sample size = 142, $\sigma = 1$). The test statistic was $Z = 0.9533$, with a p value of 0.1702 (which is greater than 0.01), and $P(z \leq Z) = 0.8298$. These results indicate that 1.080 does not differ significantly from 1, suggesting that our WRMR value is equivalent to 1. This evidence supports the model's goodness of fit.

The chi-squared statistic yielded significant results because the number of sample moments (which determines the model's degrees of freedom) was excessively high. This led to an overestimation of standard errors and increased statistical sensitivity, making it more likely to detect discrepancies that could be due to chance alone. Escolà-Gascón[65] previously warned about this issue and recommended against using the chi-squared test to assess model fit in SEM. To further

examine whether the differences were random, we conducted a Z-test on the WRMR indicator, which confirmed that they were. Therefore, the significant chi-squared result may reflect random fluctuations rather than true model misfit.

The results and previous analyses confirm the econometric validity of the predictive model shown in Figure 4, indicating that recall and nonlocal perception in resuscitated patients with CA account for 32% of the variance in NDEs. This significant finding invites a reconsideration of the boundaries of conscious experience at the moment of death.

Quantum analysis using $Q_{\text{Fisher-Escolà}}$

The final part of our analysis focused on the quantum transition induced by the entanglement of the qubit states in the EM1 circuit. To develop and clarify these analyses, we began with two empirical premises:

(1) Groups A, B, and C were methodologically differentiated such that perceptual stimuli in groups A and B were exposed under quantum entanglement conditions, whereas group C was not. In other words, the collapses of the EM1 circuit in groups A and B were conditioned by previously entangled states. In contrast, group C employed the CM1 circuit, which, although it maintained quantum superposition to ensure maximum randomness, did not include entanglement effects.

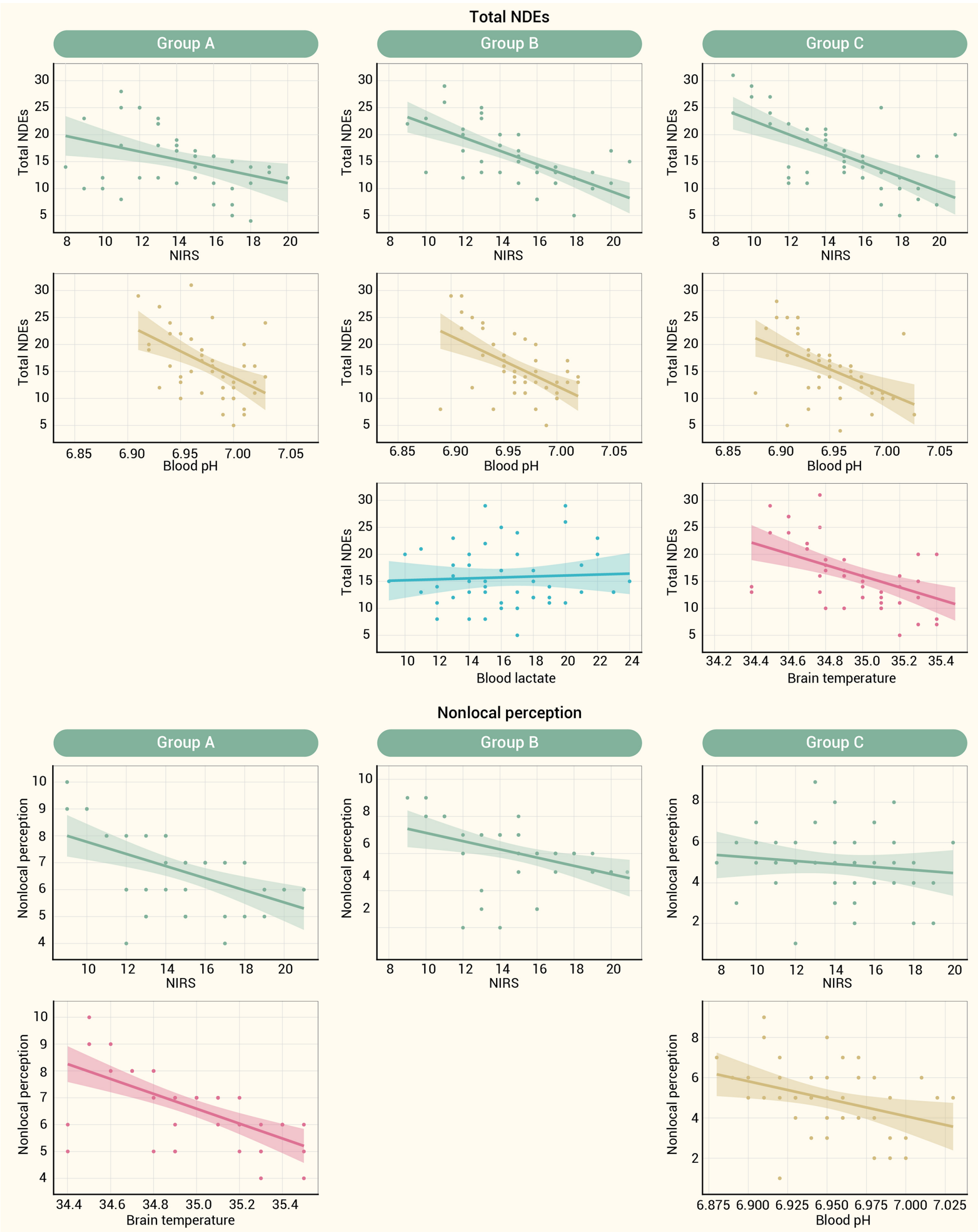

Figure 3. Predictive models of NDEs and nonlocal perception were developed using a perceptual memory recall test Auditory stimuli were presented during cardiopulmonary arrest episodes, and the memory test was administered following the patient's recovery. Near-infrared spectroscopy (NIRS) consistently emerged as a significant exogenous predictor across all groups and for both endogenous variables.

(2) Table 3 shows significant discrepancies in the means for the nonlocal perception row, which resulted in an anomalous variance of 17% (see the ω^2 coefficient).

Assuming that quantum entanglement was transferred in a way that influenced both the collapses (forming latent patterns) and the presentation of sensory stimuli, we estimated the proportion of variance explained by nonlocal perception accuracy at this stage. This integrated variance encompasses both the proportion of variance attributable to classical linear response patterns (denoted as V_k and calculated using tetrachoric matrices and the principal axis method) and the quantum-explained variance (for which we used the C_q and I_Q components, both defined in the subsection the Fisher-Escolà Q coefficient). This integrated proportion of explained variance is referred to as the $Q_{\text{Fisher-Escolà}}$ (see Equations 12 and 13).

The value of Q is modeled using the theoretical Fisher-Escolà distribution developed by Escolà-Gascón and Benito-León.[64] This theoretical distribution has been mathematically validated and is designed to determine whether observed variations in Q deviate from what would be expected by chance. The $Q_{\text{Fisher-Escolà}}$ test is designed to detect structured deviations from randomness under a specific quantum-inspired modeling framework. Still, it should not be interpreted as direct evidence of quantum information transmission or nonlocal quantum causation in conscious experience. While the results are consistent with the proposed model, they do not uniquely discriminate between quantum-derived informational structure and other forms of structured, non-random classical processes, which remain plausible alternative explanations.

In any event, this enables us to conduct a hypothesis test on the Q values obtained for groups A and B in this study. It is important to recall that the 17% variance from the ω^2 coefficient serves as a point estimator for the parameter β in the Q equation (see Equation 13). Carrying out the computations (Equation 14) for patient groups A (two factors extracted using tetrachoric correlations and principal axis factoring) and B (likewise, two factors extracted using the same method), we obtained the following results:

$$Q_{\text{Fisher-Escolà}-A} = V_k\left(1+\beta C_q(4\cdot I_Q)\right) = 0.372(1+0.170 \times 0.9997(4 \times 0.5001)) = 0.4985 \text{ and}$$

$$Q_{\text{Fisher-Escolà}-B} = V_k\left(1+\beta C_q(4\cdot I_Q)\right) = 0.389(1+0.170 \times 0.9994(4 \times 0.5002)) = 0.5212. \quad \text{(Equation 14)}$$

Applying the Fisher-Escolà modeling approach and adjusting the significance thresholds to 0.01, 0.001, and 0.0001, the probabilities that the observed Q distribution matched the theoretical $Q_{\text{Fisher-Escolà}}$ distribution (i.e.,

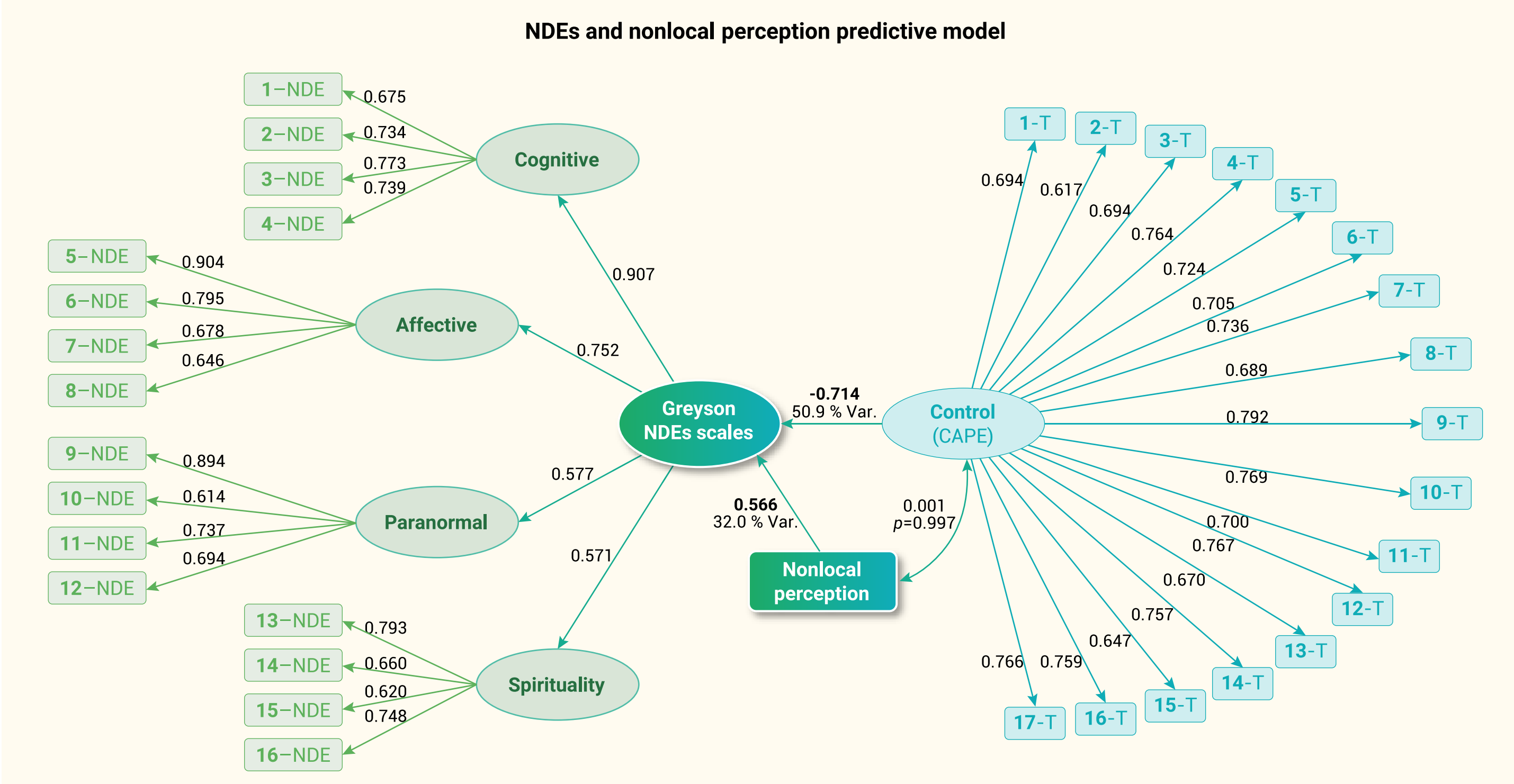

Figure 4. Path diagrams of the theoretical model analyzed in this study Structural equation modeling of latent variables was conducted to demonstrate the absence of correlation between the CAPE control subscale—associated with perceptual disturbances within the psychosis spectrum—and accuracy in nonlocal perception. The parameter estimates provide statistical confirmation that (1) perceptual disturbances represent hallucinatory experiences that are negatively correlated with NDEs, suggesting an ontologically distinct origin, and (2) while nonlocal perception shows no association with CAPE scores—supporting the view that it involves a fundamentally different perceptual mechanism than that observed in psychotic episodes—it significantly predicts up to 32% of the variance in Greyson scale scores in a negative direction. This finding is particularly noteworthy, as it supports the idea that NDEs are not only ontologically distinct from psychotic-like experiences but also associated with mechanisms consistent with quantum principles. This interpretation aligns closely with the theoretical framework guiding this study.

the p values calculated for the samples in this study using the Python analysis macros and syntax developed by Escolà-Gascón and Benito-León[64]) were as follows: for group A, $P(Q_\alpha \geq Q_{\text{Fisher-Escolà}}) = P(0.459 \geq 0.4985) \approx 0.000002 < 0.0001$, and for group B, $P(Q_\alpha \geq Q_{\text{Fisher-Escolà}}) = P(0.459 \geq 0.5212) \approx 0.000001 < 0.0001$.

Therefore, the results of this study confirm that the Q values of 0.4985 and 0.5212 predict both classical and quantum effects, as indicated by statistically significant proportions of variance that could not be explained by chance, in accordance with the theoretical $Q_{\text{Fisher-Escolà}}$ distribution. The increments (Δ) in the Q statistics for groups A and B were as follows: for A, $\Delta Q = 0.4985 - 0.3720 = 0.126$, and for B, $\Delta Q = 0.5212 - 0.3890 = 0.132$. These results were also statistically significant under the assumption $\Delta Q \sim N(0, 1)$, with p values below 0.01 in both cases. In contrast, if the calculations based on Equation 13 were applied to group C—following the mathematical rules proposed by Escolà-Gascón and Benito-León[64]—we would obtain a β parameter equal to 0, and the proportion of variance explained by the quantum entanglement effects would also be equal to 0. These results support the conclusion that significant evidence was found for a quantum transition effect that influenced participants' accuracy, allowing them to access sensory information that, according to the average scores of group C, would otherwise be neither simple nor easily accessible.

Therefore, our findings support theories positing a quantum component of conscious experience, even during pre-death states or in critical conditions such as prolonged CA. Although the present findings demonstrate a statistically robust, structured deviation from randomness, they do not, by themselves, establish quantum processes as the causal mechanism underlying the observed effects. The quantum framework adopted here should therefore be understood as a hypothesis-generating model for structured informational effects rather than as a confirmed explanation, and alternative non-quantum mechanisms capable of producing comparable structure cannot be excluded by the current design. The implications of this discovery are further discussed in the following section.

DISCUSSION

Neurophysiological correlates and biomarkers of NDEs

The regression model results revealed that NIRS was consistently present across all combinations and groups. In every model, NIRS showed a negative correlation with both NDEs and nonlocal perception. This finding aligns with existing hypotheses that conceptualize NDEs as hallucinatory consequences of the brain's multiple attempts to resist death. As cerebral oxygen levels decrease, iatrogenic effects in the nervous system emerge, activating certain neural pathways that remain active despite reduced oxygen. These pathways would maintain some degree of perceptual function—albeit altered—giving rise to NDEs. While this explanation appears plausible for the subjective components of NDEs, applying the same logic to nonlocal perception becomes harder to justify.

In the case of nonlocal perception, subjective experiences were not evaluated; instead, the focus was on verifiable hits—accurate information that the dying brain could plausibly have perceived. While we support the position of the research team led by Martial et al.,[86] our evidence suggests that even in the moments preceding final death, the brain may access information under conditions that would be classically improbable or unpredictable—though not impossible if certain quantum rules, specific to the experimental design of this study, are taken into account (see the subsection quantum functioning of nonlocal perception in NDEs for further detail). The position of Martial et al.[86] centers on exploring materialist and biophysical explanations that could scientifically account for the anomalous features attributed to NDEs. While they do not deny or dismiss the possibility that certain NDE content may occasionally correspond to actual events during resuscitation, their research focuses on identifying potential neurological markers that might explain even such coincidences. In contrast, our study directly investigates these cases using auditory identification tests and applies rigorous controls to rule out all known biases that could otherwise account for such outcomes. Within this context, we emphasize that, due to the methodological safeguards implemented in our experiment, we present evidence suggesting that consciousness may be preserved even during states of reversible clinical death.

Specifically, we show that patients—or their brains—despite undergoing prolonged CA under biological conditions typically deemed incompatible with conscious experience, appear to retain sensitivity to quantum information embedded in auditory stimuli delivered via the IBM Brisbane quantum computer. This finding calls for reconsidering the assumption that consciousness ceases during critical periods when biological life is severely compromised, and it suggests the possibility of greater continuity of conscious awareness than current science has yet acknowledged.

Interestingly, brain temperature was a significant exogenous variable for nonlocal perception in group A but not for NDEs. This qualitative distinction aligns with previous anomalous case study findings, in which individuals in deep trance states slightly lowered their body temperature, with lower levels correlating with increased anomalous perceptions, albeit not necessarily hallucinatory ones.[87] This coincidence allows us to speculate about the potential role of temperature in reducing metabolism and prolonging conscious survival beyond the cessation of minimal bodily oxygenation—a condition known as anapyrexia.[88] In fact, various studies and meta-analyses suggest that low brain temperatures may exert a protective function on certain neuroperceptual processes.[89] These findings appear consistent with those of experimental physicist Edwin May,[90] who proposed that temperature can interfere with entropy levels in physical systems—initially causing high uncertainty (i.e., elevated entropy), followed by a progressive reduction and eventual dissipation. According to Marwaha and May,[90] anomalous cognitions may emerge precisely at these moments of peak entropy, which in our case are integrated into the NDEs. We revisit this point in the subsection quantum functioning of nonlocal perception in NDEs of the discussion, where we present the reasoning and logic behind the potential quantum effects that may have occurred in our study.

In the case of NDEs, the biomarker blood lactate emerged as a significant and differential exogenous variable—but only for group B, not for the others. This positive correlation supports the physiological observations discussed in relation to the NIRS biomarker: when cells produce energy without sufficient oxygen through anaerobic glycolysis, they generate lactate. Prior research has shown that lactate concentrations are positively associated with perceptual alterations.[91]

An unexpected finding was the low correlation between the measured variable and predisposition to brain plasticity. While neuroplasticity has been associated with enhancements in certain cognitive abilities[74] and even with potential nonlocal quantum learning mechanisms,[80] it appears that in dying patients, this mechanism was not correlated with either NDEs or nonlocal perception at the time of death. This is a distinctive element: although previous research found that neuroplasticity benefits memory,[92] those studies did not involve extreme conditions, such as the moments immediately before death during prolonged CAs. Under terminal conditions—and in accordance with the thermodynamic law of system preservation[93]—it is likely that the brain's plastic capacity diminishes due to oxygen deprivation and its reliance on lactate-related energy sources. Consequently, the brain activates survival pathways aimed at preserving existing systems rather than inducing morphological adaptations characteristic of plasticity. The moment molecular structural preservation fails, cell death ensues.[94] Outside of this physiological condition, which is intrinsic to the cross-sectional value of our measurements at the point of near-death, we found no other plausible explanation for why neuroplastic predisposition was not a significant predictive variable—despite having been so in numerous other studies.

However, the most medically relevant reflection lies in the proportions of explained variance we uncovered. According to our analyses, under the most optimistic scenario, if biological determinism accounts for up to 56.8% of the variance in NDEs and nonlocal perception, what explains the remaining 43.2% of the variance in such experiences? It is important to note that we have experimentally and statistically controlled for unconscious response biases, false memories, and hallucination production. Thus, based on our analysis, we cannot determine what accounts for the remaining 43.2%. Still, we can confirm that it is not attributable to these three variables, which were successfully controlled in this study. Ruling out these known variables leaves us with 43.2% uncertainty, which is even more challenging for current medical sciences and neuroscience. This observation poses a numerical challenge to the international scientific community that, if contested, must now be refuted not through rhetoric or opinion but through new data and statistical models.

Quantum functioning of nonlocal perception in NDEs

The rules derived from quantum mathematics are grounded in the effects of latent patterns,[64] which are systematic perturbations observable through specific statistical techniques in sets of collapses involving previously entangled qubits, functioning in a bipartite manner. They are referred to as "patterns" because they reflect a structured imprint of nonlocal entanglement, as measured and demonstrated in quantum targets such as qubits. In the design of our study, because the collapses determined the exposure of sensory stimuli, any entanglement effect influencing those collapses could, through causal chaining, also affect the patients' perceptual responses (this is precisely what we controlled for through the randomized allocation of patients to the three groups). If such an impact were systematic, it would constitute the first empirical evidence demonstrating how quantum-level functioning might interfere with certain channels of nonlocal perception and, more specifically, partially explain the occurrence of NDEs with meaningful or reality-consistent content.

In relation to this point, our results indeed revealed systematic effects. Both group A and group B achieved significantly more hits than expected by chance, occurring only in the groups exposed to prior quantum entanglement between qubits. In contrast, the hit rates in group C were largely due to random fluctuations and did not exhibit systematic patterns.

These patterns, which contain structural evidence of certain quantum properties, are also described as latent because their detection is not feasible using analytical procedures grounded solely in classical statistics or standard quantum mathematics.[80] When quantum rules are applied to detect them, the problem of contextual decoherence[95] and macroscopic determinism[96] prevents the preservation of entanglement and nonlocal behavior, forcing the effects to become mechanically local, linear, or explainable by non-quantum sources (e.g., hidden variables). Similarly, when conventional statistical procedures are employed to identify these latent patterns, the primary issue is their limited power or sensitivity.[97] This leads to false negatives in scenarios where latent patterns are actually present, which undermines the external validity of any statistical inferences drawn from such analyses.

Since latent patterns do not originate from the general macroscopic reality but possess a distinct material-quantum ontology, new and specific measurement techniques were required to detect such variation. This research trajectory was initiated by Escolà-Gascón and Benito-León[64] and remains in its early stages of development. For this reason, we approach the confirmation of quantum effects in NDEs with due caution. Accordingly, the quantum interpretation advanced here should be regarded as provisional and model dependent, pending future studies that can discriminate it from alternative non-quantum sources of structured informational effects.

Avoiding potential misunderstandings. It is important to emphasize that the quantum component of our experimental design was not embedded in the physical properties of the auditory stimuli themselves but rather in the informational structures that governed their sequencing and exposure. The sounds presented during CA resuscitation were conventional acoustic stimuli, devoid of any quantum encoding. What rendered them quantum relevant was the structural configuration—derived from the collapse patterns of entangled qubits—that determined how and when each sound would be projected for each patient. In this context, entanglement did not act directly on the stimuli but rather on the organization of information that structured their delivery. This subtle yet fundamental distinction helps explain why the physiological biomarkers showed no significant variation across groups (see Table 3)—since no physical quantum stimulus was involved—and why the effect emerged primarily within the domain of subjective memory recall.

This interpretation aligns closely with the concept of latent patterns previously discussed. These patterns reflect non-obvious, embedded structures resulting from prior entanglement that, while undetectable through classical statistical methods or standard quantum measurements, can exert downstream effects on cognitive processing. In our study, the entangled informational structures functioned as latent carriers of order within the stimulus sequences. Their influence manifested not through direct sensory perception but through enhanced recognition performance in the memory task—specifically in the groups exposed to quantum-derived informational configurations. The fact that such differences were observed only in cognitive outcomes, and not in physiological markers, supports the hypothesis that certain mental functions—particularly those involved in memory reconstruction under altered

states of consciousness such as NDEs—may be sensitive to quantum-structured information. This suggests the possible existence of symbolic or representational mechanisms in the mind that are attuned to relational patterns shaped by entanglement, even in the absence of direct quantum interactions at the biological level.

Clinical implications for neurology, anesthesiology, and resuscitation medicine

Neurological implications. Recent evidence challenges the traditional view that the brain is completely inactive during clinical death. Multiple studies have documented late surges in organized brain activity, particularly high-frequency gamma oscillations, which are associated with conscious processing.[98,99] In dying patients, gamma power bursts and enhanced functional connectivity have been observed even as cardiac output sharply declines.[100] Notably, these surges concentrate in posterior cortical "hot zones" (temporo-parietal-occipital regions), which are essential for visual awareness.[101] This may provide a plausible neurophysiological basis for the vivid perceptual phenomena reported in NDEs—such as life review and the experience of light—during periods when the electroencephalogram (EEG) is expected to be isoelectric.

These findings suggest that transient hypoxia-induced excitation can briefly restore network dynamics capable of sustaining internally generated consciousness, aligning with subjective reports of clarity and lucidity during NDEs. Neurochemical factors may also modulate this process. BDNF, which supports synaptic plasticity and neuronal survival, was elevated in participants who reported more intense NDEs and successfully recalled auditory stimuli presented during CA. This raises the possibility that stress-related surges in neurotrophic signaling might extend the brain's residual cognitive window during states of clinical death, enabling memory encoding.

Taken together, these data suggest that the dying brain can exhibit complex, coherent activity consistent with conscious-like processing. NDEs may therefore represent not mere hallucinations but an extreme, transient state of disinhibited cortical activity. These insights challenge existing assumptions in neuroscience about the thresholds of consciousness at the end of life and support the implementation of cerebral monitoring, such as EEG and cerebral oximetry, during resuscitation.

Implications for anesthesiology. The emergence of lucidity under presumed unconsciousness has parallels in anesthetic practice. Intraoperative awareness—whereby patients later recall events despite receiving general anesthesia—demonstrates that consciousness can unpredictably resurface under pharmacological suppression. Although rare, with an estimated incidence of 0.1%–0.2% (or 1–2 cases per 1,000 general anesthetics) according to the American Society of Anesthesiologists Task Force on Intraoperative Awareness,[102] such episodes highlight the limitations of current monitoring techniques in detecting residual awareness.

NDEs during CA broaden this concern, showing that conscious experience may arise even during conditions thought to be incompatible with awareness (e.g., deep coma or isoelectric EEG). Anesthesiologists are thus encouraged to refine monitoring strategies, including frontal EEG and depth-of-consciousness indices, particularly in high-risk or prolonged procedures.

The parallels extend further. Certain anesthetics and psychoactive agents, especially ketamine—a dissociative NMDA antagonist—are well-documented to induce phenomenological states that closely mimic spontaneous NDEs. These include OBEs, time distortion, depersonalization, and profound tranquility or transcendence.[103–105] A large-scale semantic analysis of subjective reports has confirmed that ketamine-induced states are more similar to NDEs than those associated with serotonergic psychedelics.[104]

These similarities support the hypothesis that shared neurochemical mechanisms—such as transient NMDA receptor blockade and glutamatergic dysregulation under extreme stress—may underpin NDEs. One speculation is that the dying brain might endogenously release ketamine-like compounds or enter a disinhibited cortical state, temporarily supporting heightened internal awareness. These findings reintroduce anesthesiology into broader debates on the neural correlates of consciousness, including theories that embrace non-classical processes such as quantum brain dynamics. Notably, our study found correlations between verified quantum entanglement effects and conscious recall, lending empirical support to theoretical models—such as the Hameroff and Penrose theory[57]—that attempt to explain how awareness might emerge under otherwise suppressive conditions.

Implications for resuscitation practice

In emergency and critical care contexts, the finding that some CA survivors report awareness during CPR has both practical and ethical implications. Prospective trials such as AWARE-II have documented that up to 32% of survivors recalled lucid experiences or sensory awareness during periods of apparent clinical death.[24] Even more strikingly, EEG recordings from a subset of patients revealed re-emergent organized brain activity—spanning delta, theta, alpha, and beta bands—during ongoing CPR, which occasionally persisted for 30–60 min after the onset of CA.

These observations suggest that current clinical definitions of asystole may not fully capture meaningful brain activity. In practice, this could influence how resuscitation teams decide when to continue or cease efforts. If EEG or NIRS devices indicate residual cortical activity during CPR, clinicians may be encouraged to extend the duration of resuscitation or adopt brain-protective strategies.

Furthermore, these data raise important patient-centered considerations. If consciousness can persist during CPR, even in a small percentage of cases, sedation or analgesia protocols may need re-evaluation—particularly in resource-equipped settings. Respectful verbal communication is also warranted during resuscitation, as patients may later recall events or dialogue. This mirrors existing recommendations for comatose intensive care unit patients and is now supported by empirical NDE research.

Post-resuscitation care should include systematic inquiry into NDEs using tools such as the NDE-S by Greyson[3] and offer psychological support for those affected. While many NDEs are described as positive or transformative, some survivors struggle with existential distress, emotional volatility, or a sense of detachment from prior beliefs or relationships. Structured debriefing sessions—already recommended in some intensive care unit follow-up protocols—can help patients integrate these experiences, particularly when delivered in an open and nonjudgmental manner.

Finally, the recognition that the dying brain may retain residual consciousness prompts ethical reflection. In particular, it raises difficult questions about when death should be declared and how organ donation decisions should be timed. While current criteria for brain death remain unchanged, these findings support the view that death is a process with stages rather than an instantaneous event. Clinicians may need to acknowledge the possibility of covert consciousness during resuscitation and engage with families using language that reflects this evolving understanding.

Limitations

Although we consider the methodological quality of this study among the most robust in the field of NDEs, we acknowledge that—despite our efforts to control for biases rarely addressed in prior experimental designs—two key limitations remain. These considerations will also shape the direction of future research.

The first limitation relates to the clinical setting of intensive care. All patients who entered CA received life-saving medical intervention in accordance with standard emergency protocols. While entirely appropriate from a clinical standpoint, these interventions restricted our ability to obtain specialized electrophysiological recordings—particularly EEG—during the CA episodes. In a few cases, EEG data were available for clinical (not scientific) purposes unrelated to our study. However, we were unable to systematically apply EEG monitoring to the full sample. Several hospitals informed us that certain EEG systems could not be deployed during resuscitation without jeopardizing the effectiveness of the resuscitation. Ideally, EEG measurements in a study of this nature should be standardized across all patients to ensure the validity of statistical inference. The alternative—using different EEG systems based on hospital availability—would have required detailed methodological caveats, making this article excessively technical and lengthy. For logistical, ecological, and practical reasons, we have opted to reserve EEG analyses for a separate paper dedicated to the neurophysiological aspects of these events. While this may appear as a limitation, it did not compromise the methodological integrity or the conclusions of the present study. We believe it is essential, however, to clarify why EEG data could not be included at this stage of the project.

The second limitation opens a valuable path for further research. While NDEs appear across a broad range of cultures and belief systems,[106] certain sociological factors—beyond the scope of the current study—may be qualitatively linked to how individuals describe and interpret their NDEs. However, this limitation did not distort our current findings. In fact, the same empirical trends observed in the full sample were also present when the data were stratified by country. While this can be inferred from our ANOVA analyses, we consider it important to highlight explicitly that future studies should explore cultural variability more deeply as part of the ongoing effort to replicate and refine the hypothesis of nonlocal perception in patients undergoing CA and reporting NDEs. Specifically, we plan to examine cross-cultural differences in future analyses comparing patients who experienced CA and NDEs in England versus Spain.

CONCLUSION

At its core, this investigation into NDEs sought to rigorously address three pivotal question:

(1) Which biological and neurophysiological markers can reliably characterize the emergence of NDEs in patients undergoing prolonged CA (>2 min)?
(2) To what extent do the brain's survival-driven mechanisms give rise to nonlocal perceptual processes (through which patients may access external information in the absence of sensory input or measurable physiological activation, and later recall such information in memory tasks, even when controlling for confounding variables such as false memories, hallucinations, or psychotic symptoms)?
(3) Can the implementation of quantum effects—specifically through computational entanglement and the latent patterns we have delineated—modulate nonlocal perception in such a way that patients exhibit enhanced clarity in recalling environmental stimuli presented during their CA?

Our findings provide empirical support for all three hypotheses while reinforcing prior theoretical frameworks and empirical data put forth by other scholars:

(1) NDEs constitute genuine phenomena and are not reducible to mere perceptual distortions within the psychosis spectrum.
(2) Nonlocal perceptual phenomena do occur during NDEs and, while currently regarded as anomalous, remain insufficiently explained by the prevailing paradigms of biomedical science.
(3) Quantum-level processes—particularly those involving pre-established entanglement—appear to enhance the accuracy of environmental recall, and such enhancement may be meaningfully interpreted through the theory of latent patterns proposed by Escolà-Gascón and Benito-León.[64]
(4) Although our conclusions do not invoke any supernatural mechanisms, they do mark a significant epistemological shift with clear clinical ramifications for the fields of anesthesiology, resuscitative medicine, and consciousness research.

The most clinically relevant implications are as follows:

(1) Residual brain activity may persist beyond clinical death, requiring reconsideration of how and when we define death, particularly during CPR and organ donation protocols.
(2) Conscious awareness can emerge during states traditionally considered unconscious, such as anesthesia and CA, which challenge current monitoring and management approaches.
(3) Integration of cerebral monitoring tools (e.g., EEG and NIRS) into resuscitation protocols could guide real-time clinical decisions and help identify patients with residual or covert consciousness.
(4) Post-resuscitation care should include screening for NDEs and offering psychological support, as these experiences can have lasting emotional and existential consequences.
(5) Anesthesiology should explore why certain brains remain conscious under adequate pharmacologic suppression, which could inform safer depth-of-anesthesia monitoring.
(6) These findings invite interdisciplinary collaboration to refine neurobiological models of consciousness, particularly at life-death boundaries, and support a more compassionate, personalized approach to end-of-life care.

In sum, the results of this study compel a reexamination of where we draw the boundaries between life and death, as well as of the brain's capacity for perceptual continuity in the face of biological cessation. NDEs appear to emerge not simply as the byproducts of a system shutting down, but as structured responses—perhaps even adaptive ones—occurring at the threshold between a life that ends and an as-yet-uncharacterized state of awareness. To be clear, we make no scientific claim regarding the existence of life after death. Any such assertion, in the context of an empirical report, would be speculative and philosophically imprecise. However, it would be equally disingenuous to ignore the evidence suggesting that previously unrecognized biological processes are activated during the dying process—processes that may function as last-resort survival mechanisms and which can be modeled using principles derived from quantum dynamics, particularly those assessed through latent pattern detection.

Should these mechanisms prove to be robust and reproducible, they would imply that the biological limits of life are more elastic than currently defined by clinical or conventional physiological standards. In doing so, they raise foundational questions about what it truly means to exist—and what, ultimately, constitutes the phenomenon we call "being alive."

RESOURCE AVAILABILITY

Materials availability

This study did not generate new unique materials/reagents.

Data and code availability

The data supporting the results of this study are available from the first corresponding author upon reasonable request. Access will be granted to qualified researchers following appropriate ethical review and verification of the absence of conflicts of interest.

FUNDING AND ACKNOWLEDGMENTS

This research was supported, through J.B.-L., by the National Institutes of Health (NINDS #R01 NS39422 and R01 NS094607) and by the Recovery, Transformation, and Resilience Plan of the Spanish Ministry of Science and Innovation (grants TED2021-130174B-C33, NETremor, and PID2022-1385850B-C33, Resonate). This publication was funded by the project PID2022-135850B-C33 supported by MCIN/AEI/10.13039/501100011033 and by ERDF "A way of making Europe." The authors of this manuscript would like to express their heartfelt gratitude to all the healthcare professionals who generously dedicated their time and effort to this study. We are equally grateful to the patients, their families, and the hospital administrators for their trust and generosity, which made this project possible. Without the altruistic support of these individuals and institutions, this research would not have been feasible. We extend our special thanks to Prof. Stanley Krippner for his trust, encouragement, and the resources he made available to help move this project forward. Our sincere appreciation also goes to Dr. David del Rosario and Mr. Xavier Ginesta of the Institute of Advanced Neuroscience of Barcelona (INAB), who kindly gave us the opportunity to share this research in a plenary session at the Science of Consciousness Conference—an event attended by leading figures in the field, including Nobel laureates. Thank you for your friendship and confidence; your support has been a meaningful source of motivation for us. Finally, we would also like to thank Mr. Alejandro Agudo for his careful review and valuable corrections to this manuscript. His sharp and discerning eye significantly contributed to refining both the clarity of the text and the overall quality of the report.

AUTHOR CONTRIBUTIONS

A.E.-G. conceived and designed the study, developed the methodology, conducted the investigation and formal analyses, curated and validated the data, prepared the visualizations, and wrote the original draft and revised versions of the manuscript. K.D. contributed to project administration and management, preparation of study materials, visualization, investigation, and provision of resources. A.D. contributed to the investigation and project administration by providing resources, preparing and curating the sample data, and

conducting a bibliographic review. N.D. supported the writing of the manuscript, provided methodological supervision, and validated the scientific content of the report. J.B.-L. supervised data preparation, contributed to writing both the original and revised versions of the manuscript, and secured funding for the project's proper development. All authors discussed the results and approved the final version of the manuscript.

DECLARATION OF INTERESTS

The authors declare that they have no conflicts of interest pertaining to this study, its findings, or the resources utilized throughout the research process.

SUPPLEMENTAL INFORMATION

It can be found online at https://doi.org/10.1016/j.xinn.2026.101355.

REFERENCES

1. Greyson, B. (2023). The darker side of near-death experiences. *J. Sci. Explor.* **37**:683–698. DOI:10.31275/20232843
2. Moody, R.A. (1975). Life after Life (Bantam Books)
3. Greyson, B. (1983). The near-death experience scale: Construction, reliability, and validity. *J. Nerv. Ment. Dis.* **171**:369–375. DOI:10.1097/00005053-198306000-00007
4. Greyson, B. (2000). Near-Death Experiences. In Varieties of Anomalous Experience: Examining the Scientific Evidence, E. Cardeña, S.J. Lynn, and S. Krippner, eds. (American Psychological Association), pp. 315–352
5. Peinkhofer, C., Dreier, J.P. and Kondziella, D. (2019). Semiology and mechanisms of near-death experiences. *Curr. Neurol. Neurosci. Rep.* **19**:62. DOI:10.1007/s11910-019-0983-2
6. Martial, C., Cassol, H., Laureys, S. et al. (2020). Near-death experience as a probe to explore (disconnected) consciousness. *Trends Cognit. Sci.* **24**:173–183. DOI:10.1016/j.tics.2019.12.010
7. Beauregard, M., Courtemanche, J. and Paquette, V. (2009). Brain activity in near-death experiencers during a meditative state. *Resuscitation* **80**:1006–1010. DOI:10.1016/j.resuscitation.2009.05.006
8. Green, J.T. (1995). Lucid dreams as one method of replicating components of the near-death experience in a laboratory setting. *J. Near-Death Stud.* **14**:49–59
9. Lindsay, N., Tassell-Matamua, N., O'Sullivan, L. et al. (2025). Trauma or transcendence? The relationship between near-death experiences and dreaming. *Dreaming* **35**:1–17. DOI:10.1037/drm0000278
10. French, C.C. (2001). Dying to know the truth: Visions of a dying brain, or false memories? *Lancet* **358**:2010–2011. DOI:10.1016/S0140-6736(01)07133-1
11. Cook, E.W., Greyson, B. and Stevenson, I. (1998). Do near-death experiences provide evidence for the survival of human personality after death? Relevant features and illustrative case reports. *J. Sci. Explor.* **12**:377–406
12. Woollacott, M., Roe, C.A., Cooper, C.E. et al. (2022). Perceptual phenomena associated with spontaneous experiences of after-death communication: Analysis of visual, tactile, auditory and olfactory sensations. *Explore* **18**:423–431. DOI:10.1016/j.explore.2021.02.006
13. Kovoor, J.G., Santhosh, S., Stretton, B. et al. (2024). Near-death experiences after cardiac arrest: A scoping review. *Discov. Ment. Health* **4**:19. DOI:10.1007/s44192-024-00072-7
14. Evrard, R., Pratte, E. and Rabeyron, T. (2022). Sawing the branch of near-death experience research: A critical analysis of Parnia et al.'s paper. *Ann. N. Y. Acad. Sci.* **1515**:5–9. DOI:10.1111/nyas.14846
15. Parnia, S., Post, S.G., Lee, M.T. et al. (2022). Guidelines and standards for the study of death and recalled experiences of death–a multidisciplinary consensus statement and proposed Future Directions. *Ann. N. Y. Acad. Sci.* **1511**:5–21. DOI:10.1111/nyas.14740
16. Martial, C., Gosseries, O., Cassol, H. et al. (2022). Studying death and near-death experiences requires neuroscientific expertise. *Ann. N. Y. Acad. Sci.* **1517**:11–14. DOI:10.1111/nyas.14888
17. Roehrs, P., Fenwick, P., Greyson, B. et al. (2024). Terminal lucidity in a pediatric oncology clinic. *J. Nerv. Ment. Dis.* **212**:57–60. DOI:10.1097/nmd.0000000000001711
18. Fountain, A. (2001). Visual hallucinations: A prevalence study among hospice inpatients. *Palliat. Med.* **15**:19–25. DOI:10.1191/026921601674921626
19. Silva, T.O., Ribeiro, H.G. and Moreira-Almeida, A. (2024). End-of-life experiences in the dying process: Scoping and mixed-methods systematic review. *BMJ Support. Palliat. Care* **13**:624–640. DOI:10.1136/spcare-2022-004055
20. Blanke, O. (2004). Out of body experiences and their neural basis. *BMJ* **329**:1414–1415. DOI:10.1136/bmj.329.7480.1414
21. French, C.C. (2005). Near-death experiences in cardiac arrest survivors. *Prog. Brain Res.* **150**:351–367. DOI:10.1016/s0079-6123(05)50025-6
22. Fritz, P., Lejeune, N., Cassol, H. et al. (2023). Near-Death Experiences: What Do We Know? In Coma and Disorders of Consciousness, C. Schnakers and S. Laureys, eds. (Springer), pp. 287–306
23. Mays, R. and Mays, S. (2024). Near-death experiences are caused by the separation of consciousness from the body: An NDE scale analysis. *J. Sci. Explor.* **38**:190–211. DOI:10.31275/20243317
24. Parnia, S., Keshavarz Shirazi, T., Patel, J. et al. (2023). Awareness during resuscitation - II: A multi-center study of consciousness and awareness in cardiac arrest. *Resuscitation* **191**:109903. DOI:10.1016/j.resuscitation.2023.109903
25. Martial, C., Fritz, P., Cassol, H. et al. (2024). Phenomenological memory characteristics and impact of near-death experience in critically ill survivors: Observations at discharge and after a 1-year follow-up. *Int. J. Clin. Health Psychol.* **24**:100478. DOI:10.1016/j.ijchp.2024.100478
26. Alcock, J.E. (1979). Psychology and near-death experiences. *Skeptical Inq.* **3**:25–41
27. Blackmore, S. (1993). Dying to Live: Near-Death Experiences (Prometheus Books)
28. van Lommel, P. (2011). Near-Death experiences: The experience of the self as real and not as an illusion. *Ann. N. Y. Acad. Sci.* **1234**:19–28. DOI:10.1111/j.1749-6632.2011.06080.x
29. Martial, C., Charland-Verville, V., Dehon, H. et al. (2018). False memory susceptibility in coma survivors with and without a near-death experience. *Psychol. Res.* **82**:806–818. DOI:10.1007/s00426-017-0855-9
30. Parnia, S. (2014). Death and consciousness–an overview of the mental and cognitive experience of death. *Ann. N. Y. Acad. Sci.* **1330**:75–93. DOI:10.1111/nyas.12582
31. Kellehear, A. (2017). Unusual perceptions at the end of life: Limitations to the diagnosis of hallucinations in Palliative Medicine. *BMJ Support. Palliat. Care* **7**:238–246. DOI:10.1136/bmjspcare-2015-001083
32. Thomas, D. and O'Connor, G. (2024). Exploring near death experiences with Children Post Intensive Care: A Case Series. *Explore* **20**:443–449. DOI:10.1016/j.explore.2023.11.003
33. Betty, L.S. (2006). Are they hallucinations or are they real? the spirituality of deathbed and near-death visions. *Omega (Westport)* **53**:37–49. DOI:10.2190/188Q-BHFK-Q7DM-JHR4
34. Greyson, B. (2022). Persistence of attitude changes after near-death experiences: Do they fade over time? *J. Nerv. Ment. Dis.* **210**:692–696. DOI:10.1097/nmd.0000000000001521
35. Parnia, S. (2024). Lucid Dying: The New Science Revolutionizing How We Understand Life and Death (New York: Grand Central Publishing)
36. Long, J. and Woollacott, M. (2024). Long-term transformational effects of near-death experiences. *Explore* **20**:103030. DOI:10.1016/j.explore.2024.103030
37. Escolà-Gascón, Á. and Rusiñol Estragues, J. (2022). Scrutinizing the relationship between subjective anomalous experiences and psychotic symptoms. *J. Sci. Explor.* **36**:24–38. DOI:10.31275/20222413
38. Escolà-Gascón, Á. (2020). Researching unexplained phenomena: Empirical-statistical validity and reliability of the Multivariable Multiaxial Suggestibility Inventory-2 (MMSI-2). *Heliyon* **6**:e04291. DOI:10.1016/j.heliyon.2020.e04291
39. Escolà-Gascón, Á. (2020). Researching unexplained phenomena II: new evidences for anomalous experiences supported by the Multivariable Multiaxial Suggestibility Inventory-2 (MMSI-2). *Curr. Res. Behav. Sci.* **1**:100005. DOI:10.1016/j.crbeha.2020.100005
40. Van Lommel, P., Van Wees, R., Meyers, V. et al. (2001). Near-death experience in survivors of cardiac arrest: a prospective study in the Netherlands. *Lancet* **358**:2039–2045. DOI:10.1016/s0140-6736(01)07100-8
41. Hess, G. (2019). Physicalism, supernaturalism, and near-death experiences: A phenomenological perspective. *J. Conscious. Stud.* **26**:86–106
42. Nelson, K.R. (2014). Near-Death experience: Arising from the borderlands of consciousness in crisis. *Ann. N. Y. Acad. Sci.* **1330**:111–119. DOI:10.1111/nyas.12576
43. Lake, J. (2019). The near-death experience (NDE) as an inherited predisposition: Possible genetic, epigenetic, neural and symbolic mechanisms. *Med. Hypotheses* **126**:135–148. DOI:10.1016/j.mehy.2019.03.016
44. Tressoldi, P., Álvarez, A.A., Facchin, N. et al. (2022). Shared death experiences: A multicultural survey. *Am. J. Hops. Palliat. Care* **40**:79–86. DOI:10.1177/10499091221093270
45. Greyson, B. (2010). Implications of near-death experiences for a postmaterialist psychology. *Psychol. Relig. Spirit.* **2**:37–45. DOI:10.1037/a0018548
46. Schwartz, S.A. (2023). Physicalist materialism: The dying throes of an inadequate paradigm. *Aust. J. Parapsychol.* **23**:139–167
47. Chalmers, D. (1995). Facing up to the problem of consciousness. *J. Conscious. Stud.* **2**:200–219
48. Fenwick, P. (2012). Can Near-Death Experiences Contribute to the Debate on Consciousness? In Exploring Frontiers of the Mind-Brain Relationship, A. Moreira-Almeida and F. Santana Santos, eds. (Cham: Springer), pp. 245–258
49. Walach, H., Tressoldi, P. and Pederzoli, L. (2016). Mental, behavioural and physiological nonlocal correlations within the generalized quantum theory framework. *Axiomathes* **26**:313–328. DOI:10.1007/s10516-016-9290-6
50. Neppe, V.M. and Close, E.R. (2015). The concept of relative non-locality: Theoretical implications in consciousness research. *Explore* **11**:102–108. DOI:10.1016/j.explore.2014.12.007
51. Wahbeh, H., Radin, D., Cannard, C. et al. (2022). What if consciousness is not an emergent property of the brain? observational and empirical challenges to materialistic models. *Front. Psychol.* **13**:955594. DOI:10.3389/fpsyg.2022.955594
52. von Lucadou, W. (1990). Non-Locality in Complex Systems. In Self-Steering and Cognition in Complex Systems: Toward a New Cybernetics, F. Heylighen, E. Rossel, and F. Demeyere, eds. (Gordon and Breach Science Publishers), pp. 65–88
53. von Lucadou, W. (1995). The model of pragmatic information (MPI). *Eur. J. Parapsychol.* **11**:58–75
54. Gernert, D. (2006). Pragmatic information: Historical exposition and general overview. *Mind Matter* **4**:141–167
55. Grinberg-Zylberbaum, J. (1981). The transformation of neuronal activity into conscious experience: The syntergic theory. *J. Soc. Biol. Syst.* **4**:201–210. DOI:10.1016/S0140-1750(81)80036-X
56. Grinberg-Zylberbaum, J. (1997). Ideas about a new psychophysiology of consciousness: The syntergic theory. *J. Mind Behav.* **18**:443–458
57. Hameroff, S. and Penrose, R. (2014). Consciousness in the universe. *Phys. Life Rev.* **11**:39–78. DOI:10.1016/j.plrev.2013.08.002

58. Walach, H., Horan, M., Hinterberger, T. et al. (2020). Evidence for anomalistic correlations between human behavior and a random event generator: Result of an independent replication of a micro-PK experiment. *Psychol. Conscious. Theory Res. Pract.* **7**:173–188. DOI:10.1037/cns0000199
59. Kauffman, S.A. and Radin, D. (2023). Quantum aspects of the brain-mind relationship: A hypothesis with supporting evidence. *Biosystems* **223**:104820. DOI:10.1016/j.biosystems.2022.104820
60. Escolà-Gascón, Á. (2024). Our brains sense the future through a new quantum-like implicit learning mechanism. *Brain Res. Bull.* **216**:111048. DOI:10.1016/j.brainresbull.2024.111048
61. Woollacott, M. (2024). Near-death experience: Memory recovery during hypnosis. *Explore* **20**:103036. DOI:10.1016/j.explore.2024.103036
62. Utts, J. (1996). An Assessment of the Evidence for Psychic Functioning. *J. Sci. Explor.* **10**:3–30
63. Grinberg-Zylberbaum, J., Delaflor, M. and Arellano, M.S. (1993). Human communication and the electrophysiological activity of the brain. *Subtle Energies Energy Med. J. Arch.* **3**:25–43
64. Escolà-Gascón, Á. and Benito-León, J. (2025). Mathematical proof of the Fisher-Escolà Q statistical distribution in Quantum Consciousness Modeling. *Comput. Struct. Biotechnol. J.* **30**:41–58. DOI:10.1016/j.csbj.2025.04.025
65. Escolà-Gascón, Á. (2022). Handbook of Statistics: Step-by-step Mathematical Solutions (New York: McGraw-Hill Educatiojn)
66. Andersen, L.W., Holmberg, M.J., Berg, K.M. et al. (2019). In-hospital cardiac arrest. *JAMA* **321**:1200–1210. DOI:10.1001/jama.2019.1696
67. Nolan, J.P., Soar, J., Smith, G.B. et al. (2014). Incidence and outcome of in-hospital cardiac arrest in the United Kingdom national cardiac arrest audit. *Resuscitation* **85**:987–992. DOI:10.1016/j.resuscitation.2014.04.002
68. Mermin, N.D. (1990). Extreme quantum entanglement in a superposition of macroscopically distinct states. *Phys. Rev. Lett.* **65**:1838–1840. DOI:10.1103/physrevlett.65.1838
69. De Fabritiis, P., Roditi, I. and Sorella, S.P. (2023). Mermin's inequalities in Quantum Field theory. *Phys. Lett. B* **846**:138198. DOI:10.1016/j.physletb.2023.138198
70. Bell, J.S. (1964). On the einstein podolsky rosen paradox. *Phys. Phys. Fiz* **1**:195–200. DOI:10.1103/PhysicsPhysiqueFizika.1.195
71. Kuno, Y. (2024). Study of quantum nonlocality by CHSH function and its extension in disordered fermions. *J. Phys. Condens. Matter* **36**:505401. DOI:10.1088/1361-648X/ad7cb4
72. Burchett, D. and Ben-Porath, Y.S. (2019). Methodological considerations for developing and evaluating response bias indicators. *Psychol. Assess.* **31**:1497–1511
73. Hasani, F., Masrour, M., Khamaki, S. et al. (2025). Brain-Derived Neurotrophic Factor (BDNF) as a Potential Biomarker in Brain Glioma: A Systematic Review and Meta-Analysis. *Brain Behav.* **15**:e70266. DOI:10.1002/brb3.70266
74. Oyovwi, M.O., Ogenma, U.T. and Onyenweny, A. (2025). Exploring the impact of exercise-induced BDNF on neuroplasticity in neurodegenerative and neuropsychiatric conditions. *Mol. Biol. Rep.* **52**:140. DOI:10.1007/s11033-025-10248-1
75. Pacciolla, A. (1996). The near-death experience: A study of its validity. *J. Near-Death Stud.* **14**:179–185
76. Lange, R., Greyson, B. and Houran, J. (2015). Using computational linguistics to understand near-death experiences: Concurrent validity for the Near Death Experience Scale. *Psychol. Conscious. Theory Res. Pract* **2**:79–89. DOI:10.1037/cns0000040
77. Stefanis, N.C., Hanssen, M., Smirnis, N.K. et al. (2002). Evidence that three dimensions of psychosis have a distribution in the general population. *Psychol. Med.* **32**:347–358. DOI:10.1017/s0033291701005141
78. Ros-Morente, A., Vilagra-Ruiz, R., Rodriguez-Hansen, G. et al. (2011). Process of adaptation to Spanish of the Community Assessment of Psychic Experiences (CAPE). *Actas Esp. Psiquiatr.* **39**:95–105
79. Fonseca-Pedrero, E., Paino, M., Lemos-Giráldez, S. et al. (2012). Validation of the Community Assessment Psychic Experiences-42 (CAPE-42) in Spanish college students and patients with psychosis. *Actas Esp. Psiquiatr.* **40**:169–176
80. Escolà-Gascón, Á. (2025). Evidence of quantum-entangled higher states of consciousness. *Comput. Struct. Biotechnol. J.* **30**:21–40. DOI:10.1016/j.csbj.2025.03.001
81. Wootters, W.K. (1998). Entanglement of formation of an arbitrary state of two qubits. *Phys. Rev. Lett.* **80**:2245–2248. DOI:10.1103/physrevlett.80.2245
82. Fisher, R.A. (1925). Theory of statistical estimation. *Math. Proc. Camb. Phil. Soc.* **22**:700–725. DOI:10.1017/S0305004100009580
83. Qu, S., Xu, F.-Q., Guo, B. et al. (2025). Quantum Fisher information in one-dimensional translation-invariant quantum systems: Large-N limit analysis. *Phys. Lett.* **529**:130103. DOI:10.1016/j.physleta.2024.130103
84. Inman, H.F. and Bradley, E.L. (1989). The overlapping coefficient as a measure of agreement between probability distributions and point estimation of the overlap of two normal densities. *Commun. Stat. Theor. Methods* **18**:3851–3874. DOI:10.1080/03610928908830127
85. Clemons, T.E. and Bradley, E.L. (2000). A nonparametric measure of the overlapping coefficient. *Comput. Stat. Data Anal.* **34**:51–61. DOI:10.1016/S0167-9473(99)00074-2
86. Martial, C., Fritz, P., Gosseries, O. et al. (2025). A neuroscientific model of near-death experiences. *Nat. Rev. Neurol.* **21**:297–311. DOI:10.1038/s41582-025-01072-z
87. Celebi, T.B., Shamulzai, A., Muraca, J. et al. (2024). Hypothermia, hallucinations, and atrial fibrillation secondary to diffuse large B-cell lymphoma. *Cureus* **16**:e75560. DOI:10.7759/cureus.75560
88. Branco, L.G.S., Gargaglioni, L.H. and Barros, R.C.H. (2006). Anapyrexia during hypoxia. *J. Therm. Biol.* **31**:82–89. DOI:10.1016/j.jtherbio.2005.11.020
89. Arrich, J., Schütz, N., Oppenauer, J. et al. (2023). Hypothermia for neuroprotection in adults after cardiac arrest. *Cochrane Database Syst. Rev.* **5**:CD004128. DOI:10.1002/14651858.cd004128.pub5
90. Marwaha, S.B. and May, E.C. (2015). Rethinking extrasensory perception. *Sage Open* **5**:2158244015576056. DOI:10.1177/2158244015576056
91. Coco, M., Buscemi, A., Ramaci, T. et al. (2020). Influences of blood lactate levels on cognitive domains and physical health during a sports stress. Brief review. *Int. J. Environ. Res. Publ. Health* **17**:9043. DOI:10.3390/ijerph17239043
92. Jäger, R., Abou Sawan, S., Orrú, M. et al. (2025). Paraxanthine enhances memory and neuroplasticity more than caffeine in rats. *Exp. Brain Res.* **243**:8. DOI:10.1007/s00221-024-06954-0
93. Elitzur, A.C. (1992). Locality and Indeterminism Preserve the Second Law. *Phys. Lett.* **167**:335–340. DOI:10.1016/0375-9601(92)90268-Q
94. Cui, C., Jiang, X., Wang, Y. et al. (2024). Cerebral hypoxia-induced molecular alterations and their impact on the physiology of neurons and dendritic spines: A comprehensive review. *Cell. Mol. Neurobiol.* **44**:58. DOI:10.1007/s10571-024-01491-4
95. Tegmark, M. (2000). Importance of quantum decoherence in Brain Processes. *Phys. Rev. E* **61**:4194–4206. DOI:10.1103/PhysRevE.61.4194
96. Vaidman, L. (2014). Quantum theory and determinism. *Quantum Stud, Math. Found.* **1**:5–38. DOI:10.1007/s40509-014-0008-4
97. Velázquez-Abad, L. (2012). Principles of classical statistical mechanics: A perspective from the notion of complementarity. *Ann. Phys.* **327**:1682–1693. DOI:10.1016/j.aop.2012.03.002
98. Borjigin, J., Lee, U., Liu, T. et al. (2013). Surge of neurophysiological coherence and connectivity in the dying brain. *Proc. Natl. Acad. Sci. USA* **110**:14432–14437. DOI:10.1073/pnas.1308285110
99. A Shaw, N. (2024). The gamma-band activity model of the near-death experience: A critique and a reinterpretation. *F1000Res.* **13**:674. DOI:10.12688/f1000research.151422.2
100. Mashour, G.A., Lee, U., Pal, D. et al. (2024). Consciousness and the dying brain. *Anesthesiology* **140**:1221–1231. DOI:10.1097/aln.0000000000004970
101. Xu, G., Mihaylova, T., Li, D. et al. (2023). Surge of neurophysiological coupling and connectivity of gamma oscillations in the dying human brain. *Proc. Natl. Acad. Sci. USA* **120**:e2216268120. DOI:10.1073/pnas.2216268120
102. American Society of Anesthesiologists Task Force on Intraoperative Awareness (2006). Practice advisory for intraoperative awareness and brain function monitoring: A report by the American Society of Anesthesiologists Task Force on Intraoperative Awareness. *Anesthesiology* **104**:847–864. DOI:10.1097/00000542-200604000-00031
103. Vlisides, P.E., Bel-Bahar, T., Nelson, A. et al. (2018). Subanaesthetic ketamine and altered states of consciousness in humans. *Br. J. Anaesth.* **121**:249–259. DOI:10.1016/j.bja.2018.03.011
104. Martial, C., Cassol, H., Charland-Verville, V. et al. (2019). Neurochemical models of near-death experiences: A large-scale study based on the semantic similarity of written reports. *Conscious. Cognit.* **69**:52–69. DOI:10.1016/j.concog.2019.01.011
105. Hack, L.M., Zhang, X., Heifets, B.D. et al. (2023). Ketamine's acute effects on negative brain states are mediated through distinct altered states of consciousness in humans. *Nat. Commun.* **14**:6631. DOI:10.1038/s41467-023-42141-5
106. Belanti, J., Perera, M. and Jagadheesan, K. (2008). Phenomenology of near-death experiences: A cross-cultural perspective. *Transcult. Psychiatry* **45**:121–133. DOI:10.1177/1363461507088001

The Innovation, Volume *7*

Supplemental Information

Quantum evidence of nonlocal consciousness during clinical death

Álex Escolà-Gascón, Kenneth Drinkwater, Andrew Denovan, Neil Dagnall, and Julián Benito-León

The Innovation, Volume *7*

Supplemental Information

Quantum evidence of nonlocal consciousness during clinical death

Álex Escolà-Gascón, Kenneth Drinkwater, Andrew Denovan, Neil Dagnall, and Julián Benito-León

Appendix

1. Operational summary of the applied controls

As outlined in the preceding subsections, the design of this study successfully controlled for the following types of bias:

1) Experimenter bias: controlled through the implementation of a double-blind protocol.
2) False memory fabrication bias: addressed by testing the parametric hypothesis concerning the mean number of correct responses in groups A and B. If this type of bias was absent, one would expect the equality hypothesis to hold, such that the difference in means between groups A and B would be zero (H_0: $\mu^A - \mu^B = 0$).
3) Unconscious response bias: addressed by comparing the fit between the distributions of correct responses in the experimental group (A) and control group B, which was exposed to emotionally neutral auditory stimuli. Unlike the previous hypothesis, this analysis focused on the degree of alignment between the response patterns across groups A and B. If no adequacy bias was present, then no significant discrepancies should appear between the two distributions.
4) Causal interdependence bias: controlled by employing random assignment of patients to experimental groups. Random assignment allows for isolation and causal inference, minimizing the risk that confounding variables generate non-random effects (i.e., patterned responses) in the data.

Having accounted for the biases listed above, in order to consider whether NDEs genuinely reflect a transition from quantum effects to macroscopic reality, it becomes necessary to assess the extent to which the results of groups A and B diverged from those of group C. This can be addressed using parametric hypothesis testing on the means and variances across the groups. In the case of variance related to quantum effects, the Fisher-Escolà distribution was applied, via computation of the Q statistic. Figure A1 summarizes the group distributions and the experimental conditions assigned to each.

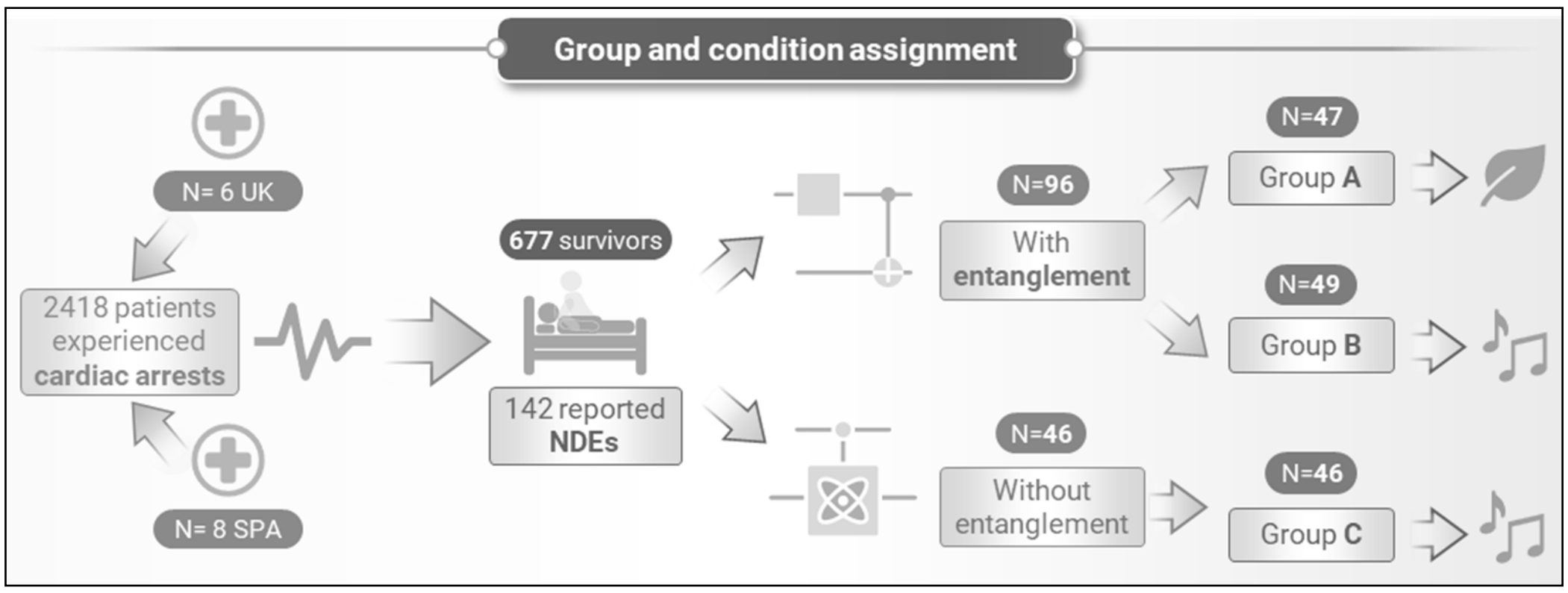

Figure A1. Process diagram for tracking, selecting, and distributing cases. It also details the three groups in this research design, the treatment conditions, and the quantum circuits used. Leaf icon= natural sounds; music icon= neutral sounds; NDE= near-death experience; SPA= Spain; and UK= United Kingdom.

Research title
Quantum evidence of nonlocal consciousness during clinical death

Authors
Álex Escolà-Gascón[1]*
Kenneth Drinkwater[2]
Andrew Denovan[3]
Neil Dagnall[2]
Julián Benito-León[4,5,6,7]*

[1]Department of Quantitative Methods and Statistics, Comillas Pontifical University, established by the Holy See, Vatican City State.
[2]Faculty of Health, Psychology and Social Care, Manchester Metropolitan University, Manchester, United Kingdom.
[3]School of Psychology, Liverpool John Moores University, Liverpool, United Kingdom.
[4]Department of Neurology, 12 de Octubre University Hospital, Madrid, Spain.
[5]Group of Neurodegenerative Diseases, Hospital Universitario 12 de Octubre Research Institute (imas12), Madrid, Spain.
[6]Centro de Investigación Biomédica en Red sobre Enfermedades Neurodegenerativas (CIBERNED), Madrid, Spain.
[7]Department of Medicine, Faculty of Medicine, Complutense University, Madrid, Spain.

***Corresponding authors**
Prof. Álex Escolà-Gascón, Ph.D., Department of Quantitative Methods and Statistics, Comillas Pontifical University, established by the Holy See, Vatican City State (office located at 23 Alberto Aguilera Street, 28015 Madrid, Spain).

Prof. Dr. Julián Benito-León, Department of Neurology, 12 de Octubre University Hospital, Madrid, Spain.

Emails
aescola@icade.comillas.edu
julianbe@ucm.es

2. Composition, illustration, and technical specifications of the quantum and classical circuits

Figures A2 and A3 in this Appendix provide the detailed graphical representations of the circuits employed in the study.

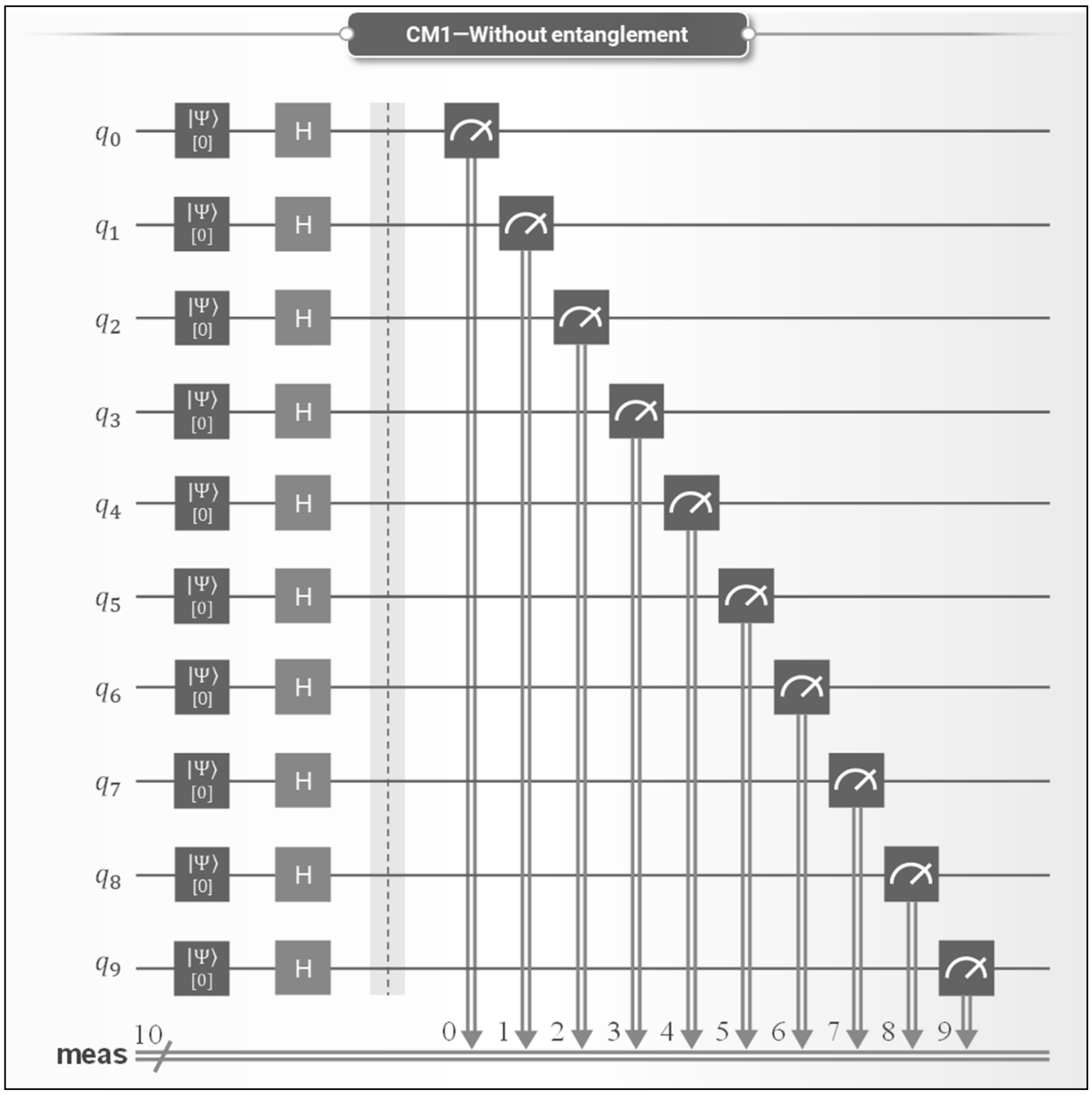

Figure A2. CM1 circuit of the control group, with no entanglement.
The Hadamard gates create superpositions that lead to fully randomized collapses.

Methodological note

The operational conditions under which these circuits were implemented are detailed in the main manuscript. In addition, the reported values of the Pauli operators correspond to sample-based estimations derived by averaging across the full set of circuit executions, in accordance with the specified sample size. The mathematical and analytical procedures employed are likewise described in detail in the main text. Here, meas 10 denotes the ten-dimensional classical measurement register, whereas labels 0 – 9 identify the corresponding classical register indices rather than fixed measurement outcomes. This summary is provided to offer a clearer overview of the scope and complexity of the analyses conducted.

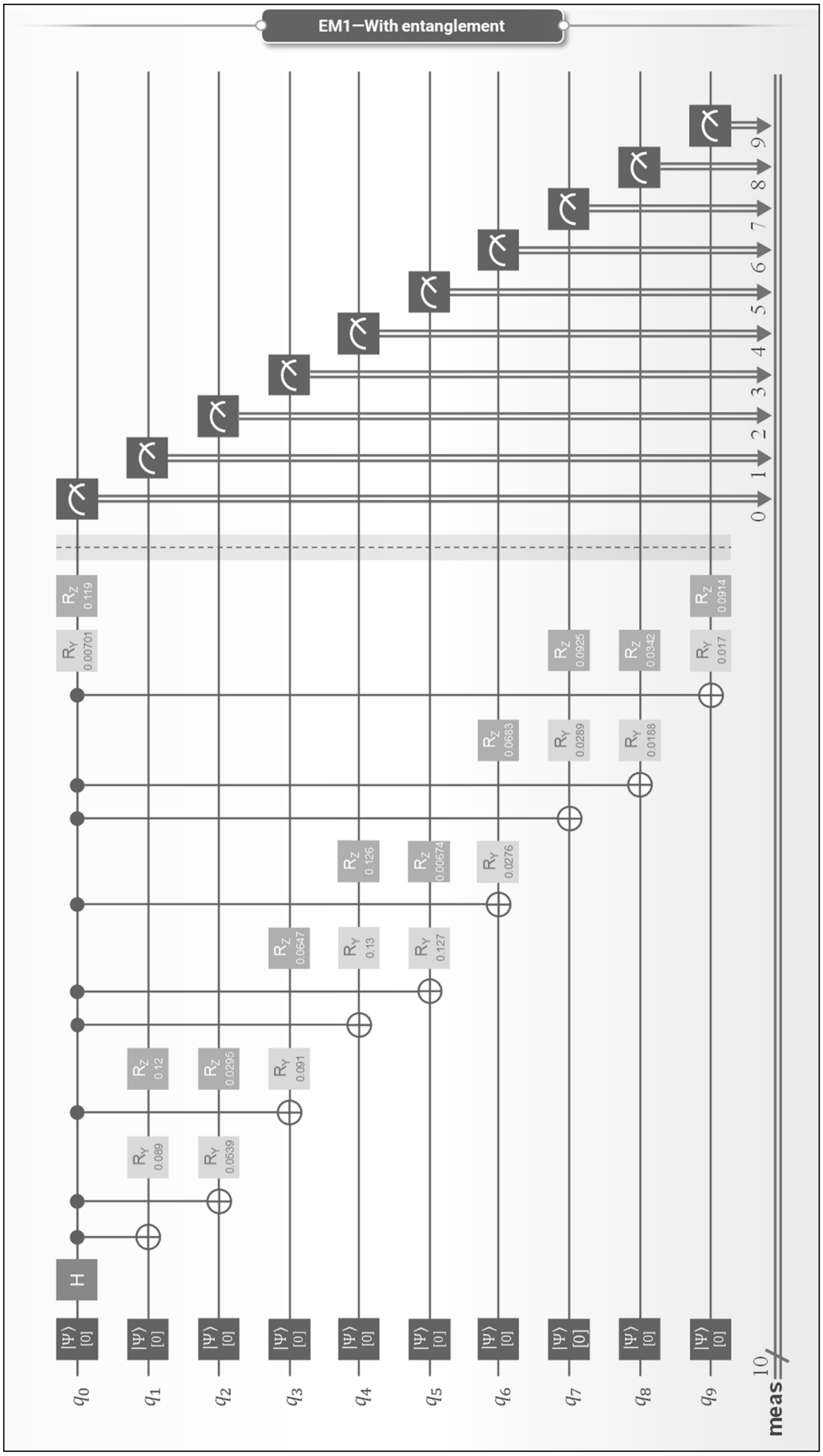

Figure A3. EM1 circuit for experimental group A, where qubits are systematically entangled using CNOT gates to generate nonlocal correlations. Rotational gates were incorporated to prevent collapse invariance.

3. Summary of hypotheses, measurements, and applied techniques

Given the extensive number of analyses and tests conducted in this study, Table A1 summarizes the relationships and correspondences among the research questions, the objects of analysis, and the mathematical-analytical tests employed. This summary is intended to provide a clearer overview of the complexity of the analyses performed.

Table A1.

Correspondences among the intended effects, the objects analyzed in relation to those effects, and the types of statistical and mathematical tests employed.

Field of analysis	Effects under investigation	Support	Types of mathematical and statistical tests applied
Medical analysis	Biomarkers associated with near-death experiences (NDEs).	Markers specified in Table 2 and scales related to NDEs.	Multiple linear regression analysis and econometric modeling.
	Biomarkers of conscious awareness during clinical death.	Average markers specified in Table 2.	Multifactorial analysis of variance, using Fisher's *F* distribution.
Quantum analysis related to consciousness	Quantum properties involved in stimulus configuration during exposure.	Density matrices obtained from the IBM supercomputer backend.	Evaluation of Mermin's GHZ inequality violations, quantum concurrence computation, and von Neumann entropy analysis.
	Empirical verification of the entanglement transition effect.	Comparison of variances and accuracy levels in the perceptual test	Multifactorial analysis of variance, using Fisher's *F* distribution.
	Gradation of the entanglement transition effect.	Explained variance using the Fisher-Escolà *Q* statistic.	Incremental analysis using the Fisher-Escolà *Q* probability distribution.
Experimental and control analysis	False memory formation biases.	Mean comparison (groups A and B) of correct responses in the perceptual test.	Multifactorial analysis of variance, using Fisher's F distribution
	Response and expectation biases.	Analysis of response sequence distributions in the perception tests.	Goodness-of-fit analysis using the *Overlap Coefficient* (OVL) and the Kolmogorov–Smirnov statistical test.
	Psychotic symptomatology and hallucinations biases.	Psychotic hallucination scale, NDE scales, and perceptual test.	Structural equation modeling of scale item responses and dimensional measures.
